# Immuno-radiotherapy enhances tumor control and induces abscopal responses in a humanized mouse model

**DOI:** 10.3389/fimmu.2026.1774955

**Published:** 2026-03-11

**Authors:** Morgane M. Cogels, Matteo Serra, Hugues Duvillier, Soizic Garaud, Laurine Verset, David Venet, Chrysanthi Iliadi, Tycho de Bakker, Laurence Buisseret, Redouane Rouas, Christos Sotiriou, Françoise Rothé, Alex de Caluwé, Dirk Van Gestel, Sébastien Penninckx

**Affiliations:** 1 Department of Radiotherapy, Institut Jules Bordet, Hôpital Universitaire de Bruxelles (HUB), Brussels, Belgium; 2 Radiophysics Laboratory, Université Libre de Bruxelles (ULB), Faculty of Medicine, Brussels, Belgium; 3 Breast Cancer Translational Research Laboratory J.-C. Heuson, Université Libre de Bruxelles (ULB), Faculty of Medecine, Brussels, Belgium; 4 Molecular Immunology Laboratory, Université Libre de Bruxelles (ULB), Faculty of Medecine, Brussels, Belgium; 5 Anatomical Pathology Center, Department of Radiotherapy, Institut Jules Bordet, Hôpital Universitaire de Bruxelles (HUB), Brussels, Belgium; 6 Cell Therapy Unit, Institut Jules Bordet, Hôpital Universitaire de Bruxelles (HUB), Brussels, Belgium

**Keywords:** immunotherapy, radiotherapy, humanized mice, abscopal effect, tumor immune microenvironment.

## Abstract

**Introduction:**

Radiation therapy (RT) offers a tool to enhance immune checkpoint inhibitor (ICI) efficacy, yet its immunomodulatory potential remains poorly understood. Here, we investigated how RT dose-fractionation regimens shape local and systemic antitumor immunity.

**Methods:**

A hematopoietic stem cell-humanized NOG mouse model was established, bearing ICI-responsive renal cell carcinoma (RCC) or ICI-resistant non-small cell lung cancer (NSCLC) and melanoma. Mice were treated with RT using different dose-fractionation regimens in combination with ICI. Tumor growth, systemic immune responses, and abscopal effects were assessed. Immune remodeling was characterized by flow cytometry, immunohistochemistry, and RNA-sequencing analyses.

**Results & discussion:**

Immuno-RT (iRT) improved tumor control across models, and induced abscopal effects in ICI-resistant models, especially in NSCLC, where 3x8 Gy combined with ICI triggered systemic responses, increased circulating monocytes and remodeled the tumor microenvironment (TME). Late-stage responses in ICI-resistant tumors were marked by low immune infiltration but enriched signatures of immune memory, cGAS/STING pathway, damage associated molecular patterns, cell death, and metabolic reprogramming. Our findings support RT as a strategy to overcome ICI resistance and validate humanized mice as a translational model for iRT research.

## 1 Introduction

Radiation therapy (RT) is a cornerstone of cancer treatment, benefiting the majority of patients throughout their disease management (1, 2). However, its efficacy is often limited by tumor resistance, recurrence, and the vicinity of radiosensitive organs at risk. In recent years, the paradigm of RT has shifted beyond its cytotoxic effects, revealing a potent capacity to shape the immune response in both positive and negative ways. This has fueled a growing interest in combining RT with immunotherapy (IO) to enhance antitumor immunity.

RT induces immunomodulation by multiple mechanisms. The induction of immunogenic cell death (ICD) leads to the release of damage-associated molecular patterns (DAMPs) such as calreticulin (CALR), heat shock protein 70 (HSP70), high mobility group box 1 (HMGB1) and ATP (3, 4), which recruit and prompt maturation and activation of antigen-presenting cells (APCs) to prime a tumor-specific immune response (5, 6). Concurrently, RT-induced DNA damage activates the cyclic GMP-AMP (cGAMP) synthase (cGAS)/stimulator of interferon genes (STING) signaling pathway (7), triggering type I interferon (IFN) release and further amplifying immune activation. Additionally, dying cancer cells release tumor-associated antigens or neoantigens (TAAs/TNAs), cytokines, and upregulate adhesion molecules on tumor vasculature (8). These mechanisms foster an environment that supports immune cell infiltration and priming of cytotoxic T cells, capable of targeting both irradiated tumors and distant unirradiated metastases, a phenomenon known as the abscopal effect (8–10). However, due to cancer immune escape properties, this effect is rarely observed with RT monotherapy. Instead, it is more commonly reported in the context of immuno-RT (iRT), where immune checkpoint inhibitors (ICIs) or other immunomodulators work in synergy with RT to overcome immunosuppression. Despite its promise, the abscopal effect remains rare in patients and its underlying mechanisms are not fully understood (11–13).

Preclinical studies have attempted to dissect the conditions required for effective iRT, exploring the role of RT dose, fractionation, tumor type, treatment volumes and sequencing. However, immunocompetent murine models have limited translational relevance due to discrepancies between murine and human immune systems, such as MHC molecules, immunoglobulins, cytokines and chemokines (14–17), and malignancies with a lack of diversity and heterogeneity in murine tumors. To bridge this gap, researchers have turned to novel humanized mouse models, providing a more relevant preclinical platform to study human immune responses to cancer treatments (18, 19).

In this study, we use a humanized mouse model to investigate the immunomodulatory effects of RT and iRT in multiple tumor models of malignancies clinically treated with ICIs and presenting different immunogenicity. Using various RT dose-fractionation regimens, we assess both local and systemic immune responses. Notably, we present the first evidence of an abscopal effect in hematopoietic stem cell (HSC)-humanized mice. Our work provides critical insights into the interplay between RT, IO and the immune system, advancing the search for effective iRT strategies.

## 2 Materials and methods

### 2.1 Cell lines and culture conditions

Human renal cell carcinoma (RCC) 786-O (VHL deficient) and human non-small cell lung carcinoma (NSCLC) H1650 (EGFR mutated and PD-L1 negative) cell lines were purchased from American Type Culture Collection (ATCC, Manassas VA, USA). Cells were cultured in RPMI-1640 supplemented with 10% fetal bovine serum (FBS), 100 U/L of penicillin, and 100 µg/mL of streptomycin (Gibco, Invitrogen, UK). The human melanoma MM043 cell line (BRAF V600E mutation) was provided by an internal biobank established from lymph node and skin metastases from patients treated at the Institut Jules Bordet. They were cultured in HAM’S F-10 medium supplemented with 5% heat-inactivated fetal calf serum, 5% heat-inactivated newborn calf serum, 200 mM of L-glutamine, 100 U/L of penicillin, and 100 µg/mL of streptomycin (all from Gibco, Invitrogen, UK). Cell lines were cultured at 37°C in humidified incubators with 5% CO_2_. All the cell lines were regularly tested for mycoplasma contamination using the MycoAlert^®^ Mycoplasma Detection Kit (Lonza, Switzerland).

### 2.2 CD34^+^ cell isolation

Fresh and frozen human cord blood samples were provided by the cord blood bank of the Université Libre de Bruxelles. Positive selection of CD34^+^ HSCs, from fresh human cord blood samples, was performed using the EasySep human whole blood CD34 positive selection kit (STEMCELL Technologies, France) and the EasyEights magnet (STEMCELL Technologies, France) according to manufacturer’s protocol. Briefly, cord blood was centrifuged with the recommended medium: phosphate-buffered saline (PBS) (Gibco, Invitrogen, UK) supplemented with 2% of fetal bovine serum (FBS) (Sigma) and 1 mM EDTA (Gibco, Invitrogen, UK). The buffy coat was recuperated and mixed with a red blood cell lysis buffer (STEMCELL Technologies, France), and the selection antibody cocktail was added (100 µL/mL of sample) followed by the RapidSpheres™. The tubes then underwent three consecutive magnet incubations: they were supplemented with the recommended medium and incubated on the magnet for 5 to 10 min at room temperature, after which the supernatant was discarded, and the bead-bound CD34^+^ cells were collected and either frozen or directly administered to the mice.

### 2.3 Mice

Five to six-week-old female NOD.Cg-Prkdc<scid> IL2rg<tm1Sug>/JicTac (NOG-F) mice were purchased from Taconic Bioscience (Denmark). Mouse housing conditions followed the applicable animal protection law. Mice were monitored at least twice weekly for body weight and general conditions. Tumor volume was measured using a digital caliper and calculated using the formula (L x W^2^)/2, where L, the length, is the longest diameter, and W, the width, is the shortest diameter. The experiments were performed according to the European Union Guidelines and validated by the local Animal Ethics Committee “Comité d’éthique du Bien-Etre Animal – Université Libre de Bruxelles (CEBEA), protocol N°: 843N.

### 2.4 Mouse humanization and tumor engraftment

Humanized mice were generated according to previously described protocols (18, 20, 21), through preconditioning with a 2 Gy whole body irradiation on a Versa HD Linac (Elekta). For the melanoma experiment, myeloablation was performed with a chemotherapeutic agent: 30 mg/kg of Busulfan (Busilvex, Pierre Faber) diluted in 0.9% saline solution injected intraperitoneally (i.p.). Twenty-four hours later, 5-10x10^4^ human umbilical cord blood-derived CD34^+^ HSCs were resuspended in stem cell culture medium (STEM CELL technologies, France) and transplanted by tail vein injection. HSCs isolated either from a single donor or as a mix of two different donors (melanoma experiment) were used.

Five weeks after HSC engraftment in NOG mice, the ratio of human CD45^+^ cells to total leukocytes in the peripheral blood was monitored by flow cytometry at fixed time intervals. Mice were considered humanized at a ratio of at least 25% (20). Accordingly, the engraftment success rate was 99% (135 mice humanized out of 137). Kinetic analysis showed that the 25% threshold for humanization was reached by week 6 with the highest level of hCD45^+^ cells observed at week 10 (60% of total leukocytes) (**Supplementary Figures 1A–D**). The average blood human immune cell populations, expressed in percentage of hCD45^+^ cells, initially showed a majority of B cells, which then decreased over time with the increase of other cell types: CD4^+^ and CD8^+^ T cells, CD14^+^ monocytes, CD56^+^ natural killer (NK) cells (**Supplementary Figure 1E**, **Supplementary Table 1**). None of the human markers were detected in non-humanized NOG mice, confirming the specificity of the staining and level of humanization reached (**Supplementary Figure 1F**).

Seven to ten weeks post HSC-engraftment, 786-O RCC, H1650 NSCLC and MM043 melanoma cell lines were engrafted subcutaneously onto one or both mouse thighs (bilaterally) synchronously according to the experimental setting. 5x10^6^ 786-O cells, or 4x10^6^ H1650 cells were engrafted per leg. For the MM043 model, the right leg (irradiated leg) was engrafted with 4x10^6^ cells and the left (unirradiated) leg with 2x10^6^ cells, to delay the reaching of tumor volume endpoint, and to mimic the clinical context, in which metastases are generally smaller than the primary tumor. Bilateral tumor settings are used as a metastatic-like model, in which RT is delivered to only one of the tumor lesions. Mice were sacrificed through an intraperitoneal injection of ketamine (100mg/kg)/xylazine (8mg/kg) followed by cervical dislocation. Sacrifices were performed once the total tumor burden (left + right tumor) reached the maximum tolerated tumor volume (2000 mm^3^).

### 2.5 Radiation planning and delivery

Humanized mice were irradiated on the right leg tumor using a PXi SmART+ X-ray irradiator (Precision X-Ray Inc., North Branford, CT, USA). The device was calibrated by an external company (Tromp Medical), ensuring robust dosimetry (periodic check + in run measurements). To facilitate treatment planning, computed tomography (CT) images were acquired using a low dose set of parameters for soft tissue imaging (40 kV, 3mA, 0.1 mm spot, 2 mm Al filter). For the treatment planning, i.e. contouring structures, setting up the radiation beams, performing dose calculations, analyzing the dose, we used the SmART-ATP software (Precision X-Ray Inc., North Branford, CT, USA). The dose (2, 5, 8 or 10 Gy) was delivered to the tumor from two angles (0 and 180 degrees) with a source to surface distance of 30 cm, at 225 kV and 13 mA, using a 0.3 mm Cu filter, via a 5 mm cylinder collimator, resulting in a dose rate of 3 Gy/min at the isocenter.

Mice were not restrained, placed on a graphite bed of 3.4 mm thickness and anesthetized by continuous isoflurane gas inhalation during the entire procedure (induction at 0.5 L/min O_2_ with 4.0% isoflurane; maintained at 0.4 L/min O_2_ with 1.5% isoflurane).

### 2.6 Systemic treatment planning and delivery

Immune checkpoint inhibitors (ICIs) were selected and administered according to adapted guidelines from published clinical studies (22–24). For RCC-bearing mice, the combination of nivolumab, an anti-programmed cell death protein 1 (PD-1) antibody (30 mg/kg, i.p.) and ipilimumab, an anti-cytotoxic T-lymphocyte-associated protein 4 (CTLA-4) antibody (10 mg/kg, i.p.) was delivered twice weekly, the former until the end of the experiment, and the latter only for two consecutive weeks. During these two weeks, both injections were performed at the same time (**Supplementary Figures 2B, D**). For NSCLC-bearing mice, pembrolizumab, an anti-PD-1 antibody (10 mg/kg, i.p.) was delivered once weekly for the duration of the experiment (**Supplementary Figure 4B**). For melanoma-bearing mice, nivolumab (30 mg/kg, i.p.) was delivered twice weekly for the duration of the experiment (**Supplementary Figure 6B**).

### 2.7 Flow cytometry on blood samples

Peripheral blood samples were collected from the facial vein into heparin-coated tubes using animal lancets (Goldenrod, Bioseb Lab Instruments, France). In brief, 70 µL of whole blood was aliquoted for the addition of different antibodies. Blood leukocytes were stained for murine CD45, human CD45, CD3, CD19, CD14, CD4, CD56 (BD Bioscience, Belgium) and CD8 (Life Technologies, Thermo Fisher Scientific, Belgium) and were then lysed with 1x Versalyse Red Blood Cell Lysis buffer (Beckman Coulter Life Science, Analis SA, Belgium). Eight-color flow cytometry acquisitions were performed on a Navios flow cytometer (Beckman Coulter) and analyzed using Kaluza Flow Cytometry Analysis v2.1 software (Beckman Coulter). The gating strategy is presented in **Supplementary Figure 1F**.

### 2.8 Immunohistochemistry staining

Excised tumors were fixed in 10% formalin before being embedded in paraffin, followed by sectioning at 4 µm using a microtome, and by hematoxylin and eosin (HE) histological staining. Immunostainings were performed with the Benchmark Ultra immunohistochemistry (IHC) system (Roche Diagnostics, Belgium) for Ki67 (1/50) (R&D Systems, Bio-Techne, Dublin), mouse CD45 (1/200), human CD45 (1/400), human FoxP3 (1/50), human CD68 (1/600), human CD86 (1/100), human CD163 (1/500) (all from Cell signaling technology, Bioké, Netherlands), human CD20 (1/200), human CD4 (1/250), human CD8 (1/500), human NCAM1 (1/200) (all from Abcam, Netherlands). Stained sections were imaged using a NanoZoomer S360MD Slide scanner system (Hamamatsu, Japan). Analysis was performed by quantifying positive cells on whole tumor slides using QuPath open source software for digital pathology image analysis (25).

### 2.9 RNA isolation and sequencing

Total RNA was isolated from paraffin-embedded tumor sections using the Qiagen RNeasy FFPE kit (Qiagen, Belgium) according to the manufacturer’s instructions. Starting from 500 ng of total RNA, strand-specific cDNA libraries were built using the TruSeq Stranded mRNA and total RNA Library Prep (Illumina, San Diego, California, United States) for paired-end sequencing. rRNA was depleted from total RNA using the RiboZero kit (Illumina, San Diego, California, United States), and sequencing was performed with a target read depth for total RNA of 50 x 10^6^ reads. RNA sequencing was performed at the BRIGHTcore sequencing facility (Université Libre de Bruxelles) using the Illumina Novaseq 6000 according to standard procedures.

### 2.10 Preprocessing of RNA sequencing data

Fastq reads were trimmed using Trimmomatic v0.39. Transcript abundance estimates were generated by Salmon v1.10.0 based on STAR v2.7.11a alignment to a compound reference consisting of the human reference genome Hg38, GENCODE release 46, plus the mouse reference mm39, GENCODE release 36. Duplicated reads were removed after alignment using the Picard tool. Transcript quantification files for human samples were imported in R (version 4.4.0) to generate gene-level expression matrices. Ensembl transcript-to-gene mappings (release 112) were retrieved via the biomaRt (version 2.60.0) package. Gene symbols were retrieved using the *biomaRt* R package and mapped to Ensembl gene IDs from the count matrices. Rows without a corresponding gene symbol were removed. To handle duplicated gene symbols, we retained the gene with the highest expression variability, as determined by standard deviation across samples. Genes with low expression (defined as fewer than 10 counts in at least three samples) were filtered out. TPM values were log-transformed (log_2_(TPM + 1)) for downstream analysis.

### 2.11 RNA sequencing analysis

Cell deconvolution was performed using *xCell* (version 1.1.0) R package. For visualization purposes, heatmaps were created using the *ComplexHeatmap* (version 2.20.0) R package on rescaled values (for both *xCell* values and gene expression). For differential gene expression analysis (DGEA), the *DESeq2* (1.44.0) R package was used. Hierarchical clustering and principal component analysis (PCA) were performed to assess sample similarity and quality check. DGEA was performed on raw counts, and genes with an adjusted *p*-value < 0.05 and an absolute log_2_ fold-change > 1 were considered differentially expressed. Volcano plots were generated to visualize differentially expressed genes (DEGs), highlighting the most significant up- and downregulated genes. Gene Set Enrichment Analysis (GSEA) was performed using the *fgsea* (version 1.30.0) R package with the Hallmark gene sets from the Molecular Signatures Database (MSigDB). To rank genes for enrichment analysis, a signed significance score was calculated as -log_10_(adjusted *p*-value) multiplied by the sign of the log_2_ fold-change. Results were visualized using bar plots, highlighting the normalized enrichment scores (NES) of significantly enriched pathways. Scripts and functions used for this analysis are available at https://github.com/BCTL-Bordet/Bulk-RNAseq-Humanized-ImmunoRT

### 2.12 Statistical analysis

Data are presented as mean +/- SEM. Graphs and statistical analyses were generated and performed using Prism 8 (GraphPad Software, USA), R (version 4.4.0) or RStudio (version 2025.05.1 + 513). Statistical significance was calculated using Student’s T test or Two-way ANOVA. Statistical significance for survival curves was calculated using the log-rank Mantel-Cox test if there were at least 10 mice per group, and the permlogrank test from the clinfun package on R if there were less than 10 mice per group. Statistical significance was considered when *p* < 0.05. For the GSEA, pathways with an adjusted *p*-value < 0.25 were considered significant.

## 3 Results

### 3.1 Immune checkpoint inhibition improves tumor control in irradiated and unirradiated RCC in a RT regimen-dependent manner by remodeling the tumor microenvironment

**Figure 1:**
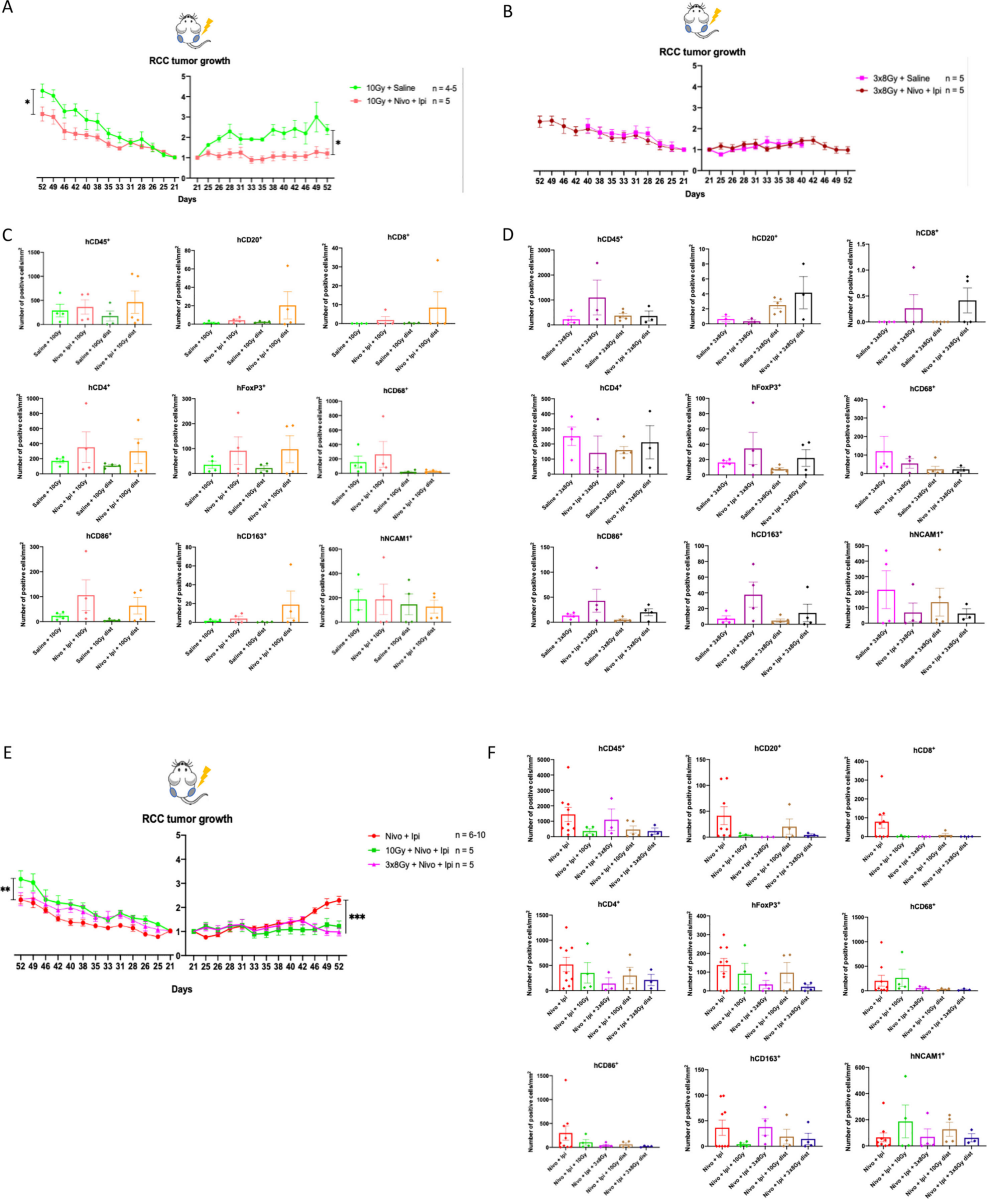
Effects of adding immunotherapy to radiotherapy in RCC tumor model: **(A, B)**. Normalized tumor growth curves of 786-O tumor-bearing humanized mice. Growth of left unirradiated tumors is displayed on the left of the Y axis, while growth of right irradiated tumors is displayed on the right of the Y axis. The X axis represents the days after cancer cell injection. **(A)** Mice are treated with 10 Gy + saline (n = 4-5) or 10 Gy + nivolumab + ipilimumab (n = 5). **(B)** Mice are treated with 3x8 Gy + saline (n = 5) or 3x8 Gy + nivolumab + ipilimumab (n = 5). Mice in the 3x8 Gy + saline treatment group were sacrificed earlier for reasons unrelated to the experimental endpoints. **(C, D)**. Immune cell density in 786-O tumors stained and detected by chromogenic immunohistochemistry. **(E)** Normalized tumor growth curves of 786-O tumor-bearing humanized mice. Growth of left unirradiated tumors is displayed on the left of the Y axis, while growth of right irradiated tumors is displayed on the right of the Y axis. The X axis represents the days after cancer cell injection. Mice are treated with nivolumab + ipilimumab (n = 6-10), 10 Gy + nivolumab + ipilimumab (n = 5) or 3x8 Gy + nivolumab + ipilimumab (n=5). **(F)** Immune cell density in 786-O tumors stained and detected by chromogenic immunohistochemistry. All data represent either individual values with mean, or mean +/- SEM. Differences between tumor growth curves were calculated using Two-way ANOVA. *p<0.05, **p<0.001, ***p<0.0001, ns not statistically significant.

To assess the effects of adding ICIs to RT compared to RT monotherapy on tumor responses, we engrafted human renal cell carcinoma (RCC) 786-O cells bilaterally in our humanized mice. The RCC model, clinically responsive to ICIs (26, 27), was used as proof of concept for its ability to respond to iRT and to undergo immune cell infiltration by the reconstituted human immune cells. The right leg tumor was irradiated with different regimens of RT (1x10 Gy or 3x8 Gy), alone or in combination with anti-PD-1 (nivolumab) and anti-CTLA-4 (ipilimumab) (**Supplementary Figures 2A, B**). Irradiated and contralateral unirradiated tumor control was enhanced by the addition of ICI compared to 1x10 Gy alone (**Figure 1A**, **Supplementary Figures 2E, F**), indicating a response to IO in this mouse model. However, 3x8 Gy alone led to complete inhibition of the irradiated tumor growth and its combination with anti-PD-1 and anti-CTLA-4 did not further improve local or distant tumor control (**Figure 1B**, **Supplementary Figures 2G, H**). Mice treated with 3x8 Gy + saline; 1x10 Gy + nivolumab + ipilimumab; and 3x8 Gy + nivolumab + ipilimumab exhibited some weight loss (**Supplementary Figure 2J**).

RCC tumors were extracted at the end of the experiment for tumor microenvironment (TME) characterization using chromogenic immunohistochemistry (IHC). Quantification of immune cell infiltration showed no significant difference between treatment groups. However, the addition of anti-PD-1 plus anti-CTLA-4 treatment following 1x10 Gy irradiation, compared to 1x10 Gy alone, tended to enhance tumor infiltration of hCD45^+^ cells, including hCD20^+^ B cells, hCD8^+^ and hCD4^+^ T lymphocytes, hFoxP3^+^ regulatory T cells, hCD68^+^ macrophages, hCD86^+^ antitumor and hCD163^+^ protumor macrophages, but did not impact hNCAM1^+^ NK cell infiltration. This increase was observed in both irradiated and unirradiated tumors (**Figure 1C**, **Supplementary Figure 3**).

Interestingly, with the 3x8 Gy regimen, ICIs did not systematically enhance immune infiltration. Only hCD8^+^, hFoxP3^+^, hCD86^+^ and hCD163^+^ cells were increased in both irradiated and unirradiated tumors of mice receiving 3x8 Gy + ICI compared to 3x8 Gy alone. Additionally, the tumor levels of infiltrated immune cells were generally lower with 3x8 Gy compared to 1x10 Gy (**Figure 1D**, **Supplementary Figure 3**).

These results suggest that anti-PD-1 plus anti-CTLA-4 improve tumor control following 1x10 Gy, both locally and at distance by promoting immune cell infiltration or retention within the TME. On the other hand, 3x8 Gy completely inhibited local tumor growth, not allowing further improvements by the addition of ICIs. Finally, the lack of response in the unirradiated tumor of mice receiving 3x8 Gy may partly be explained by potential killing of immune cells by 3x8 Gy, as suggested by tumor IHC.

### 3.2 Radiotherapy improves the control of irradiated RCC tumors *in vivo* and negatively impacts the TME

To assess the local and systemic effects of iRT compared to ICI alone in ICI-responsive tumors, humanized mice were engrafted bilaterally with RCC tumors. Mice were treated with either ICI alone, or with an iRT setup involving the local irradiation of the right tumor only (1x10 Gy or 3x8 Gy) (**Supplementary Figures 2C, D**). This setup allows the evaluation of potential abscopal effects of iRT, where iRT would induce superior tumor control in the unirradiated contralateral tumor compared to ICI alone. Both iRT regimens similarly improved local tumor control (irradiated tumor) compared to ICI alone. On the contralateral tumor, no abscopal effect was observed, and the largest tumors were in the 10 Gy + nivolumab + ipilimumab groups, suggesting a deleterious effect of the treatment (**Figure 1E**, **Supplementary Figures 2F, H, I**).

At the end of the experiment, RCC tumors were extracted for chromogenic IHC. Quantification of immune cell populations infiltrating RCC tumors showed no significant difference between treatment groups, however, the addition of RT to ICI, compared to ICI alone, tended to decrease tumor immune infiltration of both myeloid and lymphoid cells: hCD45^+^, hCD20^+^, hCD8^+^, hCD4^+^, hFoxP3^+^, hCD68^+^, hCD86^+^, hCD163^+^ cells, but not hNCAM1^+^ cells (**Figure 1F**, **Supplementary Figure 3**). This decrease was observed in both irradiated and unirradiated tumors. Both locally and at distance, 1x10 Gy preserved all immune cells better than 3x8 Gy, except for CD163^+^ protumor macrophages.

### 3.3 iRT elicits an abscopal effect in ICI-resistant models in a RT regimen-dependent manner

To investigate the potential of different RT regimens or iRT to overcome ICI-resistance, we used an ICI-resistant, EGFR-mutated and PD-L1 negative NSCLC (H1650) model. In addition, we explored the benefits of iRT in a BRAF V600E mutated melanoma (MM043) cell line, derived from the untreated metastasis of a patient at our institute, considered IO-resistant because his lesions progressed under IFNα (first FDA-approved IO) (NCT00002763).

**Figure 2:**
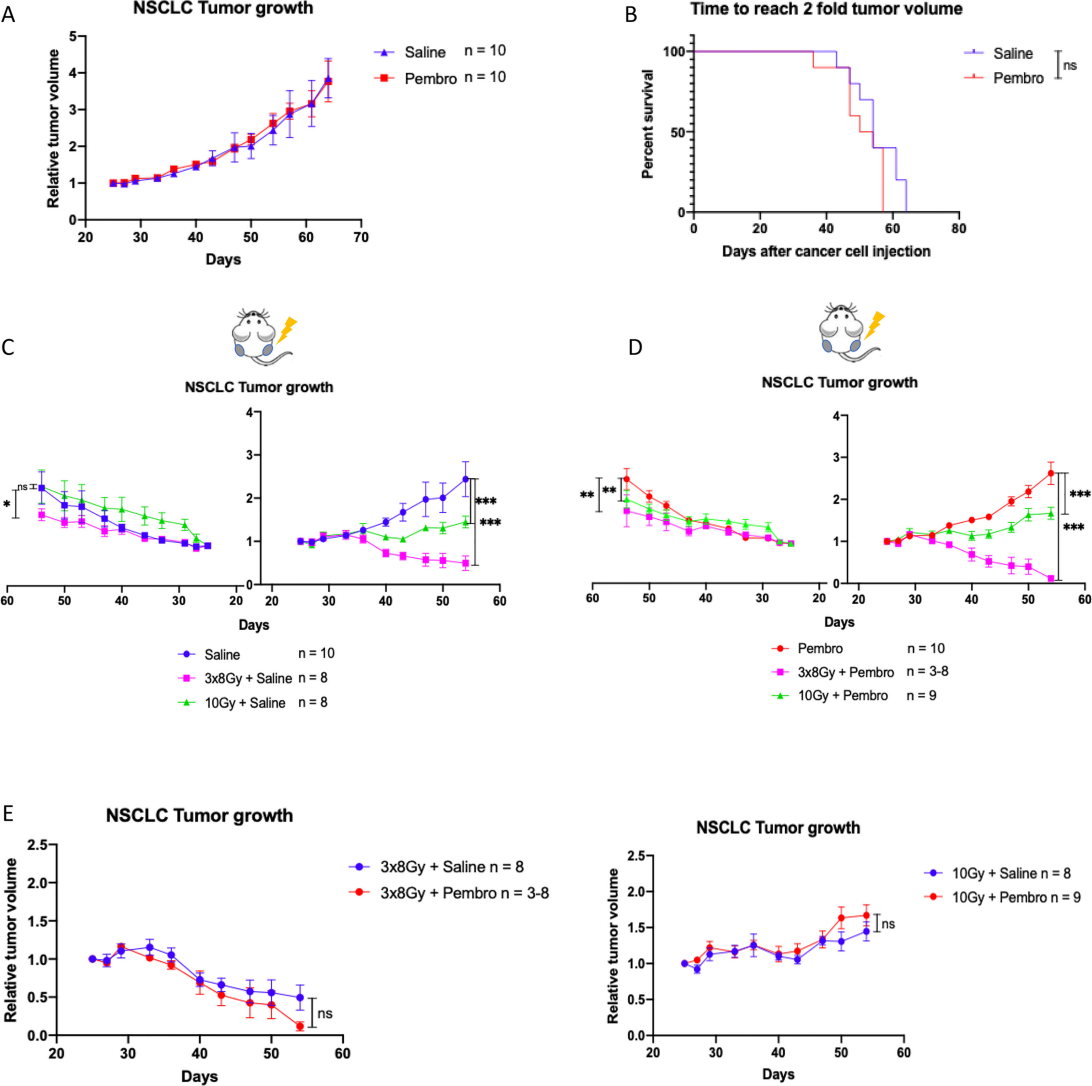
Response to iRT in NSCLC model: **(A)** Normalized tumor growth curve of H1650 tumor-bearing humanized mice. Mice are treated with saline control (n = 10), or pembrolizumab (n = 10). **(B)** Survival curve of H1650-tumor bearing humanized mice, showing total tumor burden evolution under saline control versus pembrolizumab. **(C–E)**. Normalized tumor growth curves of H1650 tumor-bearing humanized mice. Growth of left unirradiated tumors is displayed on the left of the Y axis, while growth of right irradiated tumors is displayed on the right of the Y axis. The X axis represents the days after cancer cell injection. In C mice are treated with saline control (n = 10), 3x8 Gy + saline (n = 8), or 10 Gy + saline (n=8). In D mice are treated with pembrolizumab (n = 10), 3x8 Gy + pembrolizumab (n = 3-8), 10 Gy + pembrolizumab (n=9). In E mice are treated with radiotherapy given with a saline control versus combined with pembrolizumab. All data represent mean +/- SEM. Differences between tumor growth curves were calculated using Two-way ANOVA. Survival curves were compared using the log-rank (Mantel-Cox) test. *p<0.05, **p<0.001, ***p<0.0001, ns not statistically significant.

NSCLC cells were engrafted bilaterally, and mice were treated with either a saline control, anti-PD-1 (pembrolizumab), RT (1x10 Gy or 3x8 Gy) or their combinations (**Supplementary Figures 4A, B**). Treatment with pembrolizumab alone failed to control the tumor growth, confirming that this model is resistant to ICI (**Figures 2A, B**, **Supplementary Figure 4C**). Whether given alone or in combination with ICI, 3x8 Gy elicited better local tumor control compared to 1x10 Gy, with nearly complete tumor regression in the 3x8 Gy + pembrolizumab treatment group (**Figures 2C, D**, **Supplementary Figures 4D, E**). On the unirradiated side, standalone 3x8 Gy induced a small abscopal effect, while 1x10 Gy did not (**Figure 2C**, **Supplementary Figure 4D**). When combined with ICI, both 3x8 Gy and 1x10 Gy induced unirradiated tumor growth delays, defined as abscopal effects (**Figure 2D**, **Supplementary Figure 4E**). Mice treated with 3x8 Gy + pembrolizumab and 3x8 Gy + saline exhibited some weight loss (**Supplementary Figure 4F**).

These results were confirmed in the melanoma model, in which ICI alone (nivolumab) did not delay tumor growth compared to saline control (**Supplementary Figures 5A, B** + **Supplementary Figure 6C**). In this model, iRT (1x5 Gy + ICI) also induced a contralateral tumor growth delay which may be interpreted as an abscopal response (**Supplementary Figures 5C, D** + **Supplementary Figures 6C–I**). All mice lost weight after treatment start, indicating stress related to tumor growth and animal experimentation (**Supplementary Figure 6J**).

Similar to RCC, in NSCLC (**Figure 2E**) and melanoma models (**Supplementary Figure 5E**) ICI did not further improve the control of tumors irradiated with 3x8 Gy. In contrast, in the melanoma model, ICI improved the tumor control exerted by lower single doses (1x2 Gy or 1x5 Gy) (**Supplementary Figure 5E**).

### 3.4 iRT modulates proportions of circulating immune cell populations in humanized mice

To investigate the effect of treatments on systemic immunity, we compared the different human immune cell populations present in the mouse blood before and after treatment with a focus on the NSCLC model, which demonstrated a strong systemic abscopal response. Results are displayed in **Supplementary Figures 7A–D** for RCC, and **Supplementary Figures 7E–H** for melanoma.

**Figure 3:**
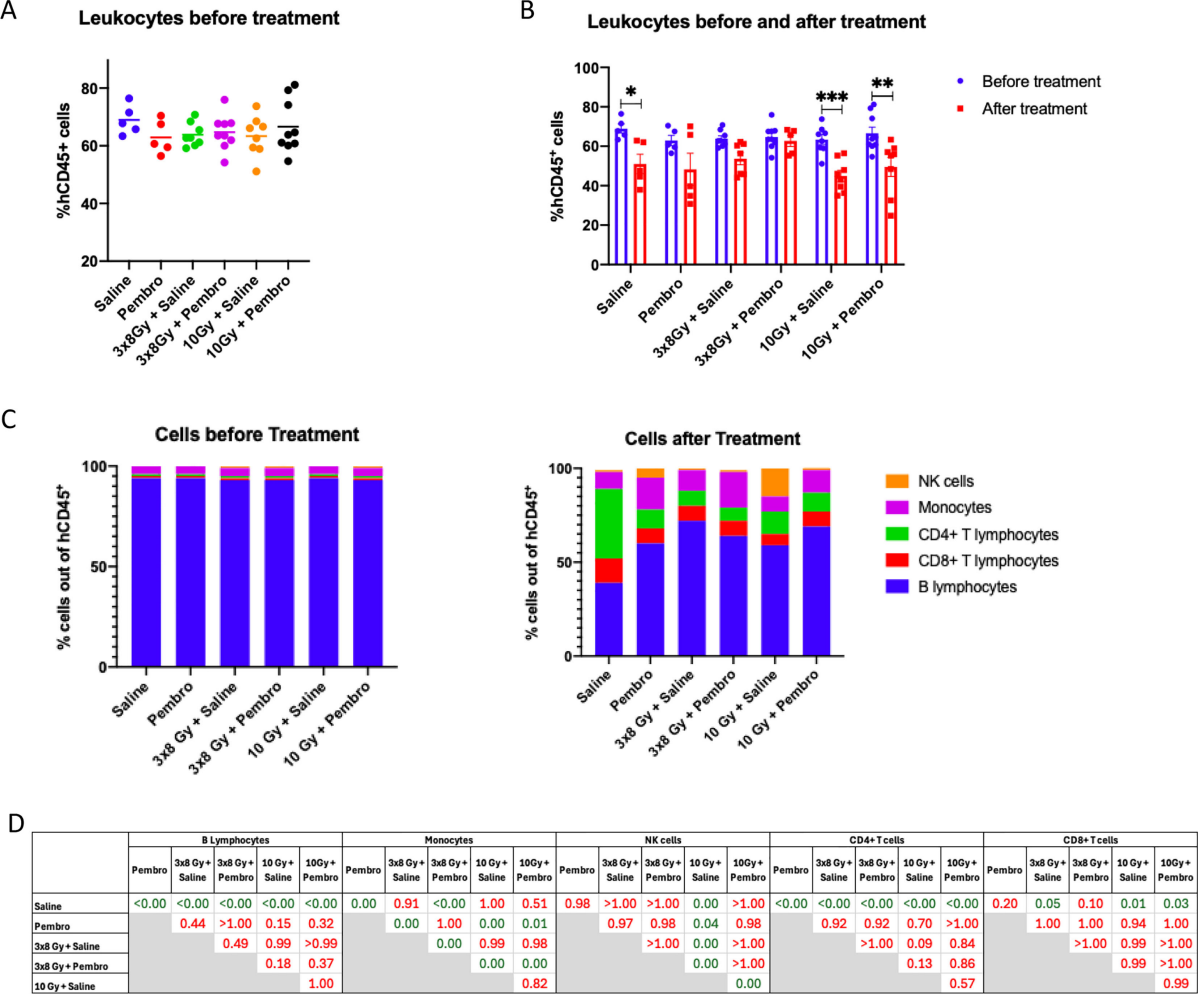
Systemic immunomodulation in NSCLC-bearing mice: **(A, B)**. Flow cytometry quantification of human CD45^+^ cells out of all leukocytes in the peripheral blood of H1650 tumor-bearing humanized mice. **(A)** shows results across groups before treatment initiation (week 10 post HSC engraftment). **(B)** shows results at week 10 (before treatment) compared to at sacrifice across treatment groups. **(C)**. Flow cytometry quantification of human CD19^+^, CD4^+^, CD8^+^, CD56^+^, and CD14^+^ cells out of human CD45^+^ cells in peripheral blood of H1650 tumor-bearing humanized mice before treatment (week 10) compared to after treatment (at sacrifice) across treatment groups. **(D)**. P-values for differences of immune cell proportions across treatment groups after treatment. All data represent either individual values with mean +/- SEM, or mean. Differences between levels of hCD45^+^ cells before and after treatment were calculated using the Mann-Whitney non-parametric T test. Differences between levels of immune cell infiltration between treatment groups were calculated using Two-way ANOVA with multiple comparisons. *p<0.05, **p<0.001, ***p<0.0001, ns not statistically significant.

In the NSCLC model, proportions of hCD45^+^ cells out of all blood leukocytes were evaluated before treatment start to ensure comparable humanization levels in all groups (**Figure 3A**). At sacrifice, overall hCD45^+^ cells decreased independently of the treatment received, although a significant decrease was observed only in the saline, 1x10 Gy + saline and 1x10 Gy + pembrolizumab groups (**Figure 3B**). Human immune cell subpopulations were then identified in each treatment group at both time points (**Figures 3C, D**). While the proportion of hCD19^+^ B cells decreased over time, all treatments significantly dampened this decrease compared to the saline control. Inversely, the proportions of hCD3^+^ T cells increased over time across treatment groups relative to before treatment, but to a lesser extent in treated groups compared to the saline control. This could suggest that all treatments either prevented T cell expansion or killed T cells in the blood or in tumors, which could be reflected by lower T cell return to the circulation. Both hCD4^+^ and hCD8^+^ T cells increased with time. Proportions of hCD56^+^ NK cells were particularly increased after 1x10 Gy + saline treatment. Proportions of hCD14^+^ monocytes were increased over time in all treatment groups, with significantly higher increases under pembrolizumab alone and in pembrolizumab + 3x8 Gy.

These results suggest that pembrolizumab and both RT regimens negatively impact circulating T lymphocytes, particularly CD4^+^ T cells, while they favor B cells and monocytes. Monocytes may thus be involved in the systemic antitumor responses, unlike B cells that are inactive in this mouse model.

### 3.5 RNA sequencing analysis shows treatment-dependent differential gene expression profiles in NSCLC tumors

To investigate modulations of the TME under the different treatments, we performed bulk RNA sequencing (RNA-seq) on 3 representative endpoint NSCLC tumor samples from saline control, 3x8 Gy + saline, 3x8 Gy + saline distant, 3x8 Gy + pembrolizumab and 3x8 Gy + pembrolizumab distant. The 3x8 Gy regimen was selected over the 1x10 Gy regimen as it induced stronger abscopal responses macroscopically both alone and when combined with ICI, which can be expected to be associated with more abscopal-related gene expression upregulation.

**Figure 4:**
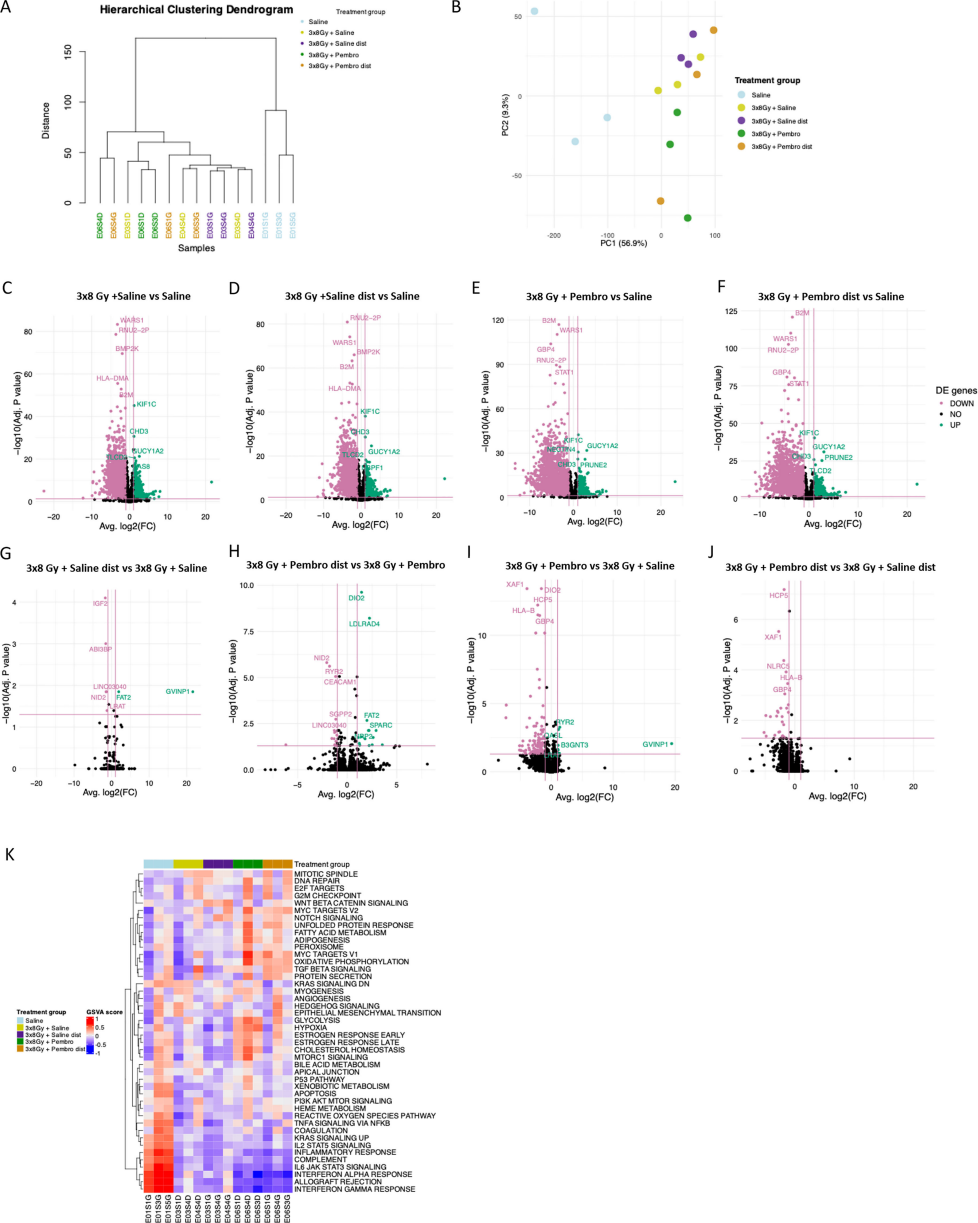
Differentially expressed genes and pathways in NSCLC tumors after treatment: Bulk RNA-sequencing was performed on sections of H1650 tumors (n = 3 biologically independent tumors per group). **(A)** Hierarchical Clustering Dendrogram showing dissimilarity and clustering between H1650 tumors of different treatment groups. **(B)** Principal Component (PC) analysis of total gene expression profiles. Each dot represents an individual tumor. **(C–J)**. Volcano plots of differentially expressed genes (DEGs). Genes with adjusted p-value <0.05 and fold change > 2 were considered differentially expressed (number of genes shown in pink and green colors indicating decreased or increased expression, respectively). **(K)** Heatmap of gene set variation analysis (GSVA) showing differential pathway activity, each column represents an individual tumor.

Differential gene expression (DGE) hierarchical clustering (**Figure 4A**) and principal component analyses (**Figure 4B**) showed clustering among tumors based on the treatment they received. Both the hierarchical clustering dendrogram and PCA plot (on the first two principal components) showed that the saline control group exhibited a distinct gene expression profile compared to the other groups.

Differential gene expression analysis showed that tumors from RT or iRT treatment groups were strongly different from the saline control group, with large numbers of genes presenting significantly (adj *p* < 0.05, abs(log_2_fc) > 1) higher and/or lower expression (**Figures 4C–F**, **Supplementary Table 2**). In contrast, fewer genes were differentially expressed when comparing the irradiated and unirradiated tumors of mice receiving RT or iRT (**Figures 4G–J**, **Supplementary Table 2**). This indicates similar profiles between irradiated and unirradiated tumors in the same treatment group (with or without pembrolizumab), and between mice receiving RT or iRT when comparing the irradiated or unirradiated tumors.

The differential gene expression analysis revealed the top 5 genes presenting higher or lower expression in different treatment groups (**Supplementary Table 2**). Interestingly, compared to their irradiated counterparts receiving RT or iRT, distal unirradiated tumors experiencing abscopal responses consistently has higher expression of tumor suppressor genes among their top 5 genes, including *SPARC* and *FAT2*.

Gene set variation analysis (GSVA) showed that several pathways presented differential activity in each treatment group. The saline control group presented elevated activity of immune-related pathways compared to treatment groups, while other pathways, including MYC targets, glycolysis, oxidative phosphorylation, fatty acid metabolism and DNA repair were enriched in tumors of mice treated with 3x8 Gy + pembrolizumab (**Figure 4K**).

Pairwise comparisons of gene set enrichment analysis (GSEA) on differentially expressed genes (DEGs) revealed that, compared to a saline control, RT and iRT downregulated most pathways both in the irradiated and contralateral unirradiated tumors, while only one pathway (myogenesis) was significantly (adj *p* < 0.25) upregulated by RT, and two pathways were significantly upregulated by iRT (myogenesis and MYC targets V2) (**Supplementary Figures 8A–D**, **Supplementary Table 3**).

In contrast, no pathways were significantly differentially expressed between irradiated and contralateral unirradiated tumors of the same mice (**Supplementary Figures 8E, F**, **Supplementary Table 3**). However, compared to RT, iRT induced significant up- and down-regulation of multiple pathways in both irradiated (**Supplementary Figure 8G**, **Supplementary Table 3**) and unirradiated tumors (**Supplementary Figure 8H**, **Supplementary Table 3**).

Strikingly, while no immune-related pathways were upregulated by treatments, they were the most numerous among significantly downregulated pathways shown by GSEA.

These results confirm the similar profiles of signaling pathways between irradiated and distant unirradiated tumors in the same treatment group. In addition, they highlight that while RT decreases most pathways when given alone, its combination with pembrolizumab increases the expression of multiple pathways compared to RT monotherapy. RT or iRT decreased tumor inflammation at the time of tumor extraction, but favored other pathways related to metabolism, cell death and stress response, regulation of cell proliferation, and tissue structure and development.

### 3.6 ICI and RT remodel the TME in NSCLC tumors

**Figure 5:**
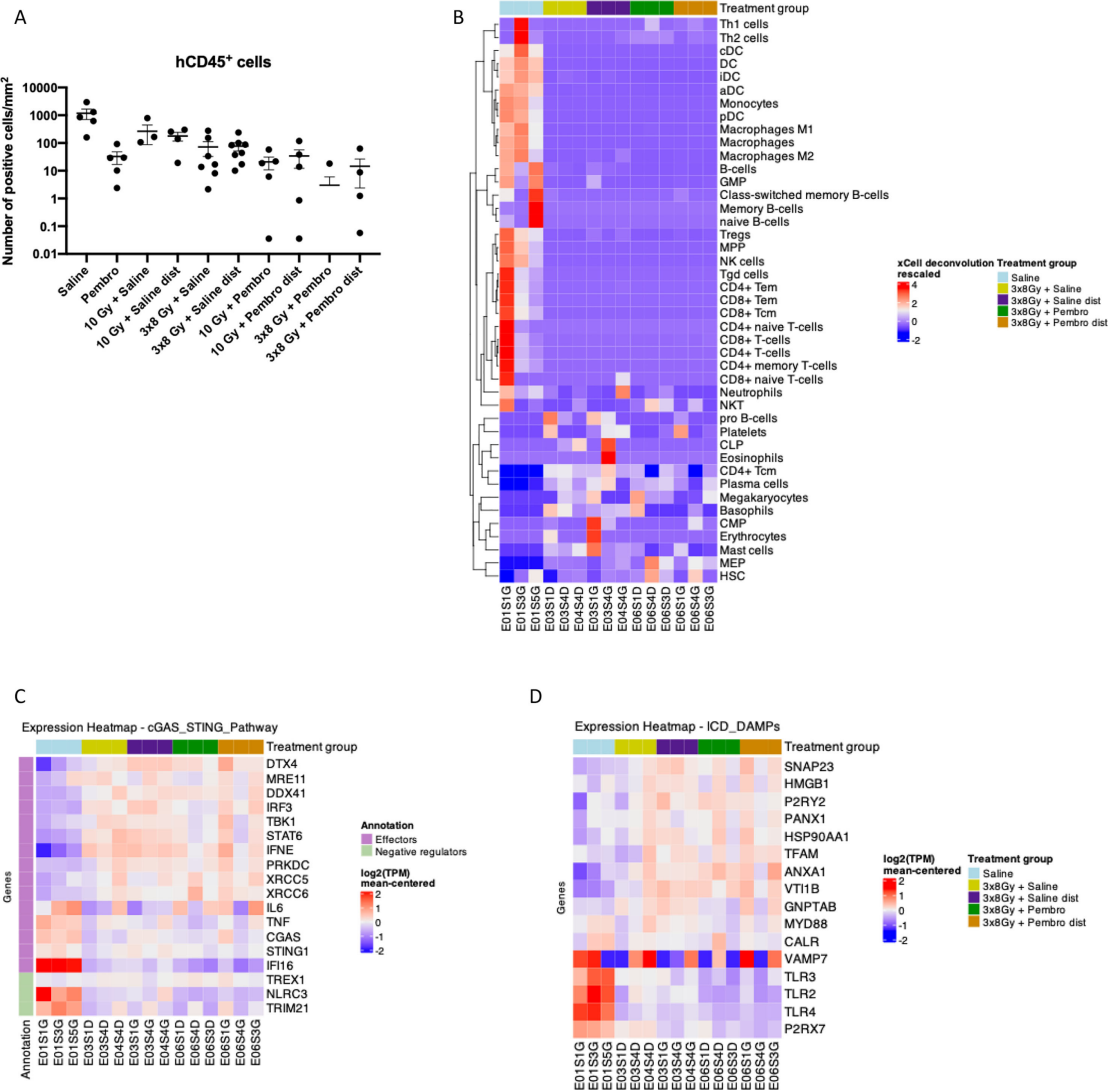
Remodeled NSCLC tumor microenvironment and immunostimulatory gene expression after treatment. **(A)** Immunohistochemistry quantification of human CD45^+^ cell density in tumors extracted from H1650 tumor-bearing humanized mice after treatment on a logarithmic scale. Data represent individual values with mean +/- SEM. Values at 0 are not represented on the graph but are considered in the mean. **(B–D)** Bulk RNA-sequencing was performed on sections of H1650 tumors (n = 3 biologically independent tumors per group). Heatmap in which each column represents an individual tumor. **(B)** Expression of human immune cell genes. **(C)** Expression of human cGAS/STING pathway-associated genes. **(D)** Expression of human damage associated molecular pattern (DAMP)-associated genes.

Chromogenic IHC on NSCLC tumors demonstrated a decrease in hCD45^+^ cell infiltration in all treatment groups compared to the saline control (**Figure 5A**), consistent with our previous observations in the DGE analysis.

Bulk RNA-seq analysis, using the xCell deconvolution package, confirmed the higher levels of human immune infiltrate in the saline-treated NSCLC tumors, and lower levels in all treatment groups (**Figure 5B**, **Supplementary Figure 9A**). However, some immune cell populations were increased in the RT-treated tumors. In particular, CD4^+^ central memory T cells were enriched in RT and pembrolizumab + RT groups. Plasma cells were increased by RT, however B cells are inactive in humanized NOG mice, thus they likely do not take part in the antitumor immune response (28–30).

### 3.7 RT and iRT induce cGAS/STING and ICD DAMP gene expression in NSCLC tumors

Treatments with RT or iRT on the tumor or on distal unirradiated tumors increased the expression of most cGAS/STING pathway-related genes compared to the saline control. Some important genes are however more highly expressed in the saline group, including: *CGAS* and *STING1* as well as DNA sensor *IFI16*, downstream effectors *IL6* and *TNF*, and negative regulators *TRIM21* and *NLRC3* (**Figure 5C**). The genes enriched in the treatment groups include genes involved in DNA sensing and STING activation (*DTX4*, *MRE11*, *DDX41*), DNA repair (*PRKDC*, *XRCC5*, *XRCC6*), and downstream effectors of the pathway (*IRF3*, *TBK1*, *STAT6*, *IFNE*). *TREX1*, encoding for the exonuclease that negatively regulates the cGAS/STING pathway is similarly expressed across saline and treatment groups.

We next evaluated the expression of genes involved in ICD, looking at DAMPs. Many DAMP-associated genes are enriched with treatments, and the majority of genes are slightly more expressed in the unirradiated tumor compared to the irradiated tumor (**Figure 5D**). We detected RNA of DAMP receptors (*TLR2*, *TLR3*, *TLR4*, *P2RX7*, *P2RY2*) mostly in the saline group, but genes of DAMPs (*HMGB1*, *TFAM*, *HSP90AA1*, *ANXA1*), DAMP secretion (*VAMP7*, *SNAP23*, *PANX1*, *VTI1B*, *GNPTAB*) and signaling (*MYD88*) were enriched or equally expressed in the treatment groups compared to the control.

These results indicate that ICD and the cGAS/STING pathway may take place in the treated tumors, consistent with the abscopal effect observed macroscopically.

### 3.8 RT and iRT induce cancer cell death and metabolism reprogramming

**Figure 6:**
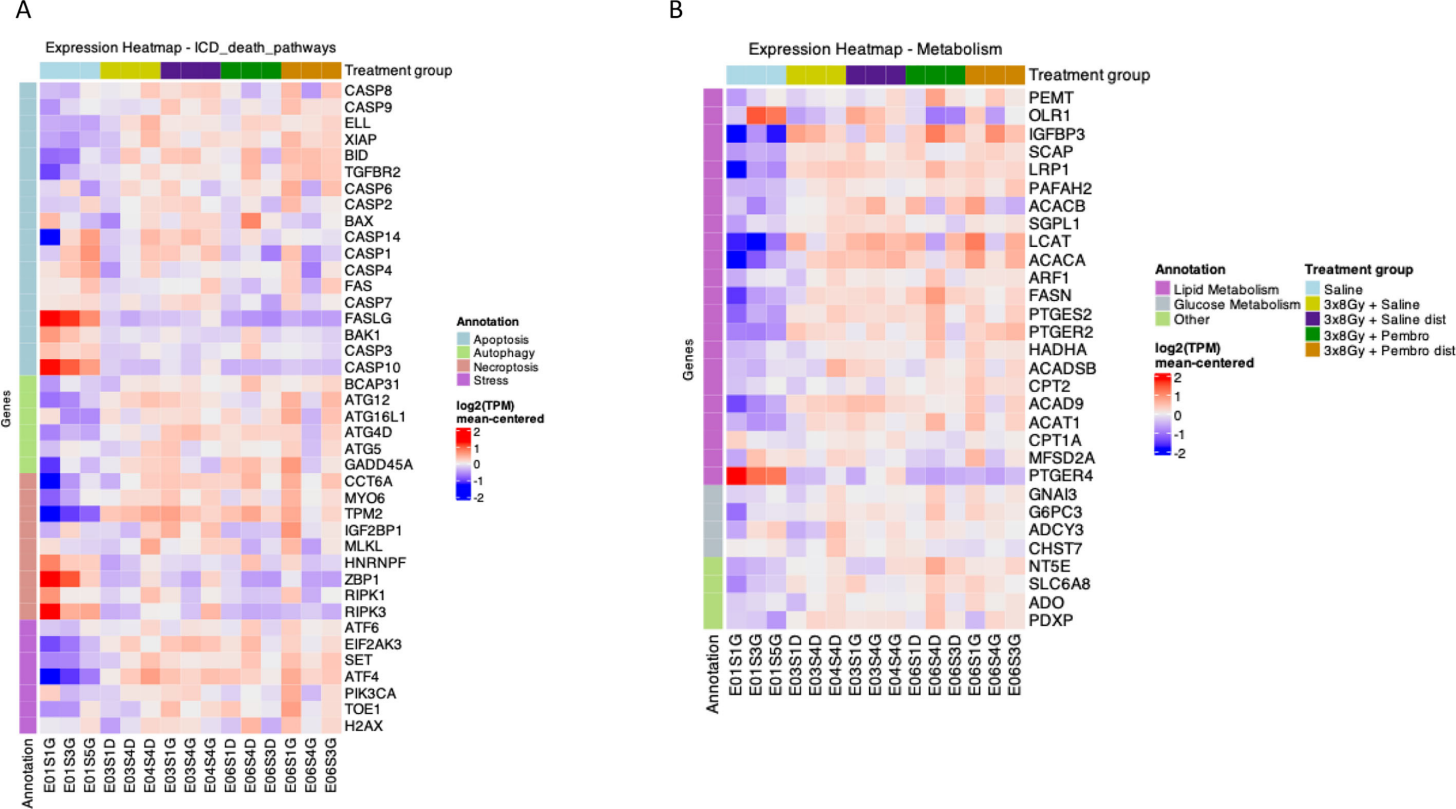
Cancer cell death and metabolic reprogramming in NSCLC tumors after treatment. Bulk RNA-sequencing was performed on sections of H1650 tumors (n = 3 biologically independent tumors per group). Heatmaps in which each column represents an individual tumor. **(A)** Expression of human cell stress and death-associated genes. **(B)** Expression of human cancer cell metabolism-associated genes.

We next investigated cell stress and cell death-related gene expression in the tumors. While some cell death-related genes were expressed in the saline control group, treatments increased the expression of most detected genes. One month after treatment start, cell death is still detectable in the tumors of treated mice. Expression of genes associated with apoptosis, autophagy, necroptosis and cell stress were evaluated (**Figure 6A**). In particular, cell stress and autophagy genes were enriched in the treated tumors compared to the saline control. Cell-death related genes were slightly enriched in the distal unirradiated tumor compared to the irradiated tumor, which may be due to the timing, with cancer cell death occurring later on the unirradiated side. Together, these results suggest that RT and iRT may delay tumor growth by inducing cancer cell death in both irradiated and unirradiated tumors.

Finally, we evaluated metabolism related gene expression, in particular fatty acid metabolism since metabolic pathways were shown in the DGEA and since tumors were previously shown to reprogram their metabolism in response to RT (31, 32). Expression of genes associated with lipid metabolism, glucose metabolism, and other metabolic pathways was evaluated, and most were enriched upon treatment compared to the saline control. Metabolic reprogramming took place both in irradiated and unirradiated tumors with or without pembrolizumab (**Figure 6B**).

## 4 Discussion

RT has emerged as a potent modulator of the immune response, particularly when combined with ICIs. Preclinical studies have shown that RT can enhance ICI efficacy and, in some cases, induce abscopal effects, in which unirradiated metastatic lesions regress (33–42). However, the underlying mechanisms and their variability across different tumor models and RT dose-fractionation regimens remain poorly understood.

In this study, we present the first evidence of an abscopal effect induced by iRT in HSC-humanized mice, a model that more closely recapitulates human immune-tumor interactions than traditional murine models, which often fail to predict clinical responses (43, 44). Previous research has demonstrated the ability of humanized mice to develop robust antitumor immune responses under ICIs, including nivolumab, ipilimumab and pembrolizumab (18, 20, 45–48). Our findings further validate their use in the context of iRT by demonstrating the ability of the reconstituted human immune system in mice to generate antitumor immune responses under iRT. By evaluating three distinct human tumor models we assessed the impact of iRT across varying immunogenic landscapes: ICI-responsive RCC (786-O) (26, 27, 49, 50), ICI-resistant NSCLC (H1650) (51–54), and IO-resistant melanoma (MM043).

Consistent with clinical observations (26, 49), the response to ICIs was tumor-type dependent: RCC tumors responded, whereas NSCLC and melanoma did not. However, the addition of RT to ICI triggered systemic antitumor responses in the ICI-resistant models, particularly in NSCLC, where a robust abscopal effect was observed. These findings, aligned with previous studies, suggest that iRT may be especially effective in tumors with intrinsic resistance to ICIs, potentially by overcoming barriers to immune priming (35, 36, 39, 55–58). In particular, our findings on the PD-L1 negative NSCLC model mirror the results of the Pembro-RT trial, in which 3x8 Gy combined with pembrolizumab specifically enhanced survival of PD-L1 negative NSCLC patients (59), which may be mediated by RT-induced PD-L1 expression on tumor cells (60).

No abscopal effects were observed in the RCC model, suggesting that in ICI-responsive models, ICIs are sufficient to elicit a durable antitumor immune response, which cannot be further enhanced by RT. In contrast, a negative abscopal effect was observed in RCC-bearing mice treated with 10 Gy + ICI, suggesting that this RT regimen promoted distal tumor growth or impaired ICI responsiveness in this model, a phenomenon previously described (61, 62).

Among the RT regimens tested, 3x8 Gy proved most effective at inducing both local control and systemic responses, particularly in NSCLC. This aligns with previous preclinical and clinical reports identifying this regimen as optimal to induce systemic antitumor immune responses (34, 35, 63–65). However, the optimal RT regimen capable of inducing abscopal responses may vary depending on the tumor type, as observed in our melanoma model and as previously shown by others (39, 42, 55, 56, 66–68). This likely reflects differences in radiosensitivity, ICI responsiveness, immunotype, oxygenation status, tumor mutational burden and other tumor-intrinsic factors. To compare the effects of fractionated and single dose RT regimens, future studies should take into account the principle of biologically effective dose (BED), and clarify if there is a link between BED values and abscopal responses. A previous study has correlated higher BED values with higher occurrence of the abscopal effect, although this study used an α/β ratio of 10 for all tumors, limiting the interpretation of its results (69).

We detected treatment-induced immunomodulation in the peripheral blood of the mice, and abscopal responses were associated with increased proportions of circulating monocytes in the NSCLC model, and T cells in the melanoma model. While T lymphocytes are expected contributors of the abscopal effect, monocytes may also play a key role by differentiating into macrophages, which have previously been implicated in this response (33). To determine the role of monocytes and T lymphocytes in the abscopal effect observed in the NSCLC and melanoma models, selective depletion of each cell type should be performed.

Importantly, we found that treatment-induced immune modulation is dynamic and not always captured at late timepoints. In RCC tumors, immune cell infiltration was increased at endpoint in both irradiated and unirradiated tumors following iRT compared to RT alone, emphasizing the immunostimulatory effects of ICI. Similar observations were recently made in a peripheral blood mononuclear cell (PBMC)-humanized mouse model bearing head and neck mucosal melanoma (HNMM) patient-derived xenografts (PDXs) (70). In contrast, immune infiltration was reduced by treatments at endpoint in the NSCLC model, despite evidence of tumor regression and systemic effects. This observation is consistent with a recent study conducted in HSC-humanized mice bearing breast cancer, where tumor immune cell infiltration did not correlate with treatment response at similar time points (57). This suggests that the immune response in ICI-resistant tumors may be transient. RT, administered only at treatment start, may have primed an immune response that was not sustained by ICI alone over time. Given the immune-resistant nature of this tumor model, effector immune cells may have retreated from tumors after exerting their cytotoxicity.

The hypothesis of a transient immune response is further supported by our bulk RNA-seq analyses on tumors at endpoint, which revealed higher transcriptional expression of memory T cells, cGAS/STING pathway and ICD DAMPs in tumors of NSCLC-bearing mice upon RT or iRT treatment. Tumor growth delay was associated with increased transcriptional expression of genes involved in apoptosis, autophagy, necroptosis and cell stress in the iRT and RT groups in both irradiated and unirradiated tumors. ICI, RT and iRT increased lipid and glucose metabolism in tumors, which has previously been associated with resistance mechanisms in response to treatment (71–73). The similarities found between transcriptional profiles of irradiated and unirradiated tumors of the same treatment group confirm again the systemic effects of RT and the presence of the abscopal effect. These findings suggest that while the immune effect may not be histologically visible at late stages, its downstream impact persists at the molecular level, supporting the presence of a temporally dynamic but impactful immune response. However, transcriptional data is not sufficient to confirm the occurrence of ICD and cell death, thus further studies are needed to confirm these findings at the protein level.

Together, our results highlight the importance of the temporal dynamics of the antitumor immune response. Relying solely on endpoint data may underestimate immune activity, as infiltration after iRT may be transient (41, 55). Humanized mice allow for extended study timeframes compared to immunocompetent models, and therefore are well suited for capturing these dynamics. Future studies incorporating serial biopsies or non-invasive techniques, such as PET or SPECT (74, 75) could more accurately monitor immune responses over time.

While HSC-humanized NOG mice likely provide a translational advantage, they do not fully recapitulate all aspects of the human immune system. Notably they present an incomplete innate immune system, lacking robust human NK and myeloid cell function. And an incomplete adaptive immune system, lacking B cell function. In addition, reconstituted human T cells are educated in the mouse thymus, limiting HLA-specific selection hence their antitumor responses are likely allogeneic (18). The observed immune response to iRT is therefore not as complex as it would be in patients, and can only provide partial mechanistic information on the abscopal effect. Despite this limitation, both human myeloid and lymphoid cells were detected infiltrating RCC tumors, and a pronounced abscopal effect was observed in the NSCLC model. This suggests that not all immune cell lineages are essential for the development of abscopal responses. Next-generation models, such as the THX mouse, which presents elevated blood levels of hCD45^+^ cells, B cells, T cells, dendritic cells, NK cells and monocytes, produces antibodies and develops a human microbiome (76), will likely provide deeper insights into the mechanisms driving iRT responses.

## 5 Conclusion

This study provides the first evidence of an abscopal effect of iRT in HSC-humanized mice and validates this mouse model for preclinical testing of iRT. In line with clinical observations, we highlight the impact of different RT regimens and tumor types on the response to iRT, with ICI-resistant tumors more likely to benefit from the combination. Moreover, our findings emphasize that immune responses are temporally dynamic, and late timepoints may not reflect the full extent of immune activation. Together, these insights highlight the need for temporal analysis of iRT effects and point toward improved strategies for selecting patients and optimizing regimens in clinical practice.

## Data Availability

The datasets presented in this study can be found in online repositories. The names of the repository/repositories and accession number(s) can be found in the article/**Supplementary Material**.

## Supplementary material

Supplementary Figure 1 **(A–D)**. Flow cytometry quantification of human CD45^+^ cells out of all leukocytes in humanized mice peripheral blood over time in individual experiments. (n = 22-44). Mice were myeloablated by sublethal irradiation before HSC engraftment **(E)**. Flow cytometry quantification of human CD19^+^, CD56^+^, CD14^+^, CD4^+^ and CD8^+^ cells out of human CD45^+^ cells over time. (n = 5). **(F)**. Representative flow cytometry plots identifying human immune subpopulations in peripheral blood of humanized and non-humanized mice. All data represent either individual values with mean or mean +/- SEM.

Supplementary Figure 2 **(A)**. Timeline for treatment of 786-O tumor-bearing humanized mice in abscopal settings. **(B)**. Treatment schedule for 786-O tumor-bearing mice. **(C)**. Timeline for treatment of 786-O tumor-bearing humanized mice in abscopal settings. Only the right tumor was irradiated when applicable, while the contralateral (left) tumor remained untreated. **(D)**. Treatment schedule for 786-O tumor-bearing mice. **(E–I)**. Individual normalized tumor growth curves (TGC) from 786-O tumor-bearing humanized mice. Growth of left unirradiated tumors is displayed on the left of the Y axis, while growth of right irradiated tumors is displayed on the right of the Y axis. The X axis represents the days after cancer cell injection. **(E)**. TGC under 10 Gy + saline. **(F)**. TGC under 10 Gy + nivolumab + ipilimumab. **(G)**. TGC under 3x8 Gy + saline. **(H)**. TGC under 3x8Gy + nivolumab + ipilimumab. **(I)**. TGC under nivolumab + ipilimumab. **(J)**. Mice weight per treatment group over time. All data represent either individual values or mean +/- SEM.

Supplementary Figure 3 **(A)**. Representative histological analysis of 786-O tumor sections stained with hematoxylin and eosin (H&E) or for immune markers in humanized mice. Tumors were treated with saline + 10 Gy (irradiated tumor), saline + 3x8 Gy (irradiated tumor), nivolumab + ipilimumab, nivolumab + ipilimumab + 10 Gy (irradiated tumor), nivolumab + ipilimumab + 3x8 Gy (irradiated tumor), saline + 10 Gy (distant unirradiated tumor), saline + 3x8 Gy (distant unirradiated tumor), nivolumab + ipilimumab + 10 Gy (distant unirradiated tumor) or nivolumab + ipilimumab + 3x8 Gy (distant unirradiated tumor). **(B)**. Quantification of human immune cell density and Ki67 expression in 786-O tumors. Data represents individual values and mean +/- SEM.

Supplementary Figure 4 **(A)**. Timeline for treatment of H1650 tumor-bearing humanized mice in abscopal settings. **(B)**. Treatment schedule for H1650 tumor-bearing mice. **(C)**. Individual tumor growth curves from H1650 tumor-bearing humanized mice under saline control (left) or pembrolizumab (right). **(D, E)**. Individual normalized tumor growth curves from H1650 tumor-bearing humanized mice. Growth of left unirradiated tumors is displayed on the left of the Y axis, while growth of right irradiated tumors is displayed on the right of the Y axis. In D mice are treated with 3x8 Gy + saline or 10 Gy + saline. In E mice are treated with 3x8 Gy + pembrolizumab or 10 Gy + pembrolizumab. **(F)**. Mice weight per treatment group over time. The X axis represents the days after cancer cell injection. Data represents individual values or mean +/- SEM. Differences between tumor growth curves were calculated using Two-way ANOVA. *p<0.05, **p<0.001, ***p<0.0001, ns not statistically significant.

Supplementary Figure 5 **(A)**. Normalized tumor growth curves of MM043 tumor-bearing humanized mice. Mice are treated with saline control (n = 4-5), or nivolumab (n = 2-7). **(B)**. Survival curve of MM043-tumor bearing humanized mice, showing total tumor burden evolution under saline control versus nivolumab. **(C–E)**. Normalized tumor growth curves of MM043 tumor-bearing humanized mice. Growth of left tumors is displayed on the left of the Y axis, while growth of right tumors is displayed on the right of the Y axis. The X axis represents the days after cancer cell injection. In C mice are treated with saline control (n = 4-5), 2 Gy + saline (n = 4-5), 5 Gy + saline (n=5), 3x8 Gy + saline (n = 6). In D mice are treated with nivolumab (n = 2-7), 2 Gy + nivolumab (n = 4-5), 5 Gy + nivolumab (n = 6), 3x8 Gy + nivolumab (n = 6). In E mice are treated with radiotherapy given with a saline control versus combined with nivolumab. All data represent mean +/- SEM. Differences between tumor growth curves were calculated using Two-way ANOVA. Survival curves were compared using the log-rank (Mantel-Cox) or permlog-rank test. *p<0.05, **p<0.001, ***p<0.0001, ns not statistically significant.

Supplementary Figure 6 **(A)**. Timeline for treatment of MM043 tumor-bearing humanized mice in abscopal settings. **(B)**. Treatment schedule for MM043 tumor-bearing mice. **(C–I)**. Individual normalized tumor growth curves from MM043 tumor-bearing humanized mice. Growth of left tumors is displayed on the left of the Y axis, while growth of right tumors is displayed on the right of the Y axis. The X axis represents the days after cancer cell injection. **(C)**. Individual tumor growth curves of MM043 tumor-bearing humanized mice under saline versus nivolumab treatment. In **(D)** mice are treated with 2 Gy + saline, in **(E)** mice are treated with 5 Gy + saline, in **(F)** mice are treated with 3x8 Gy + saline. In **(G)** mice are treated with 2 Gy + nivolumab, in **(H)** mice are treated with 5 Gy + nivolumab, in **(I)** mice are treated with 3x8 Gy + nivolumab. **(J)**. Mice weight per treatment group over time. The X axis represents the days after cancer cell injection. Data represents individual values or mean +/- SEM. Differences between tumor growth curves were calculated using Two-way ANOVA. *p<0.05, **p<0.001, ***p<0.0001, ns not statistically significant.

Supplementary Figure 7 **(A–D)**. Flow cytometry quantification of human immune cells in the peripheral blood of 786-O tumor-bearing humanized mice. **(A)** shows hCD45^+^ cells out of all leukocytes across groups before treatment initiation (at week 13 post HSC engraftment). **(B)** shows hCD45^+^ cells before treatment (week 13) compared to after treatment (week 18) across treatment groups. **(C)**. Human CD19^+^, CD4^+^, CD8^+^, CD56^+^, and CD14^+^ cells out of human CD45^+^ cells in mice peripheral blood before treatment (at week 13) compared to after treatment (at sacrifice) across treatment groups. **(D)**. P-values for differences of immune cell proportions between treatment groups after treatment. **(E–H)**. Flow cytometry quantification of human immune cells in the peripheral blood of MM043 tumor-bearing humanized mice. **(E)** shows hCD45^+^ cells out of all leukocytes across groups before treatment initiation (week 7 post HSC engraftment). **(F)** shows hCD45^+^ cells before treatment (week 7) compared to at sacrifice across treatment groups. **(G)**. Human CD19^+^, CD4^+^, CD8^+^, CD56^+^, and CD14^+^ cells out of human CD45^+^ cells in peripheral blood of MM043 tumor-bearing humanized mice before treatment (week 7) compared to after treatment (at sacrifice) across treatment groups. **(H)**. P-values for differences of immune cell proportions across treatment groups after treatment. All data represent either individual values with mean +/- SEM, or mean. Differences between levels of hCD45^+^ cells before and after treatment were calculated using the Mann-Whitney non-parametric T test. Differences between levels of immune cell infiltration between treatment groups were calculated using Two-way ANOVA with multiple comparisons. *p<0.05, **p<0.001, ***p<0.0001, ns not statistically significant.

Supplementary Figure 8 Pairwise comparison of differentially expressed pathways in NSCLC tumors: Bulk RNA-sequencing was performed on sections of H1650 tumors (n = 3 biologically independent tumors per group). **(A–H)**. Gene set enrichment analysis (GSEA) showing differentially expressed pathways. Normalized enrichment score (NSE) indicates downregulated (<0) or upregulated (>0) pathways. Adjusted p-value < 0.25 is considered significant.

Supplementary Figure 9 Remodeled NSCLC tumor microenvironment after treatment. **(A)**. Bulk RNA-sequencing was performed on sections of H1650 tumors (n = 3 biologically independent tumors per group). Heatmap in which each column represents an individual tumor, shows expression of human immune cell activation genes.

Supplementary Table 1 Levels of human immune cell populations in humanized mice peripheral blood over time.

Supplementary Table 2 Significantly differentially expressed genes in pairwise comparisons.

Supplementary Table 3 Significantly differentially expressed pathways in pairwise comparisons.

## Glossary

APC: Antigen presenting cell
CALR: Calreticulin
cGAMP: Cyclic GMP-AMP
cGAS: Cyclic GMP-AMP synthase
CT: Computed tomography
DAMPs: Damage associated molecular patterns
DC: Dendritic cell
DEG: Differentially expressed gene
DGE: Differential gene expression
DGEA: Differential gene expression analysis
GSEA: Gene set enrichment analysis
GSVA: Gene set variation analysis
HE: Hematoxylin and eosin
HMGB1: High mobility group box 1
HNMM: Head and neck mucosal melanoma
HSC: Hematopoietic stem cell
HSP70: Heat shock protein 70
i.p.: Intraperitoneally
ICD: Immunogenic cell death
ICI: Immune checkpoint inhibitor
IFN: Interferon
IHC: Immunohistochemistry
iRT: Immuno-radiotherapy
NES: Normalized enrichment scores
NK: Natural killer
NSCLC: Non-small cell lung cancer
PBMC: Peripheral blood mononuclear cell
PC: Principal component
PCA: Principal component analysis
PDX: Patient-derived xenograft
RCC: Renal cell carcinoma
RNA-seq: RNA sequencing
RT: Radiotherapy
STING: Stimulator of interferon genes
TAA: Tumor associated antigen
TGC: Tumor growth curve
TME: Tumor microenvironment
TNA: Tumor neoantigen
VST: Variance stabilizing transformation.
