# Supplementary material for: Immuno-radiotherapy enhances tumor control and induces abscopal responses in a humanized mouse model
Source: Front Immunol. 2026 Mar 11;17:1774955. doi: 10.3389/fimmu.2026.1774955 (PMC13013455; doi:10.3389/fimmu.2026.1774955)
Supplement: Supplementary file 1 [file Table1.docx]

**Supplementary Tables**

**Supplementary Table 1. Levels of human immune cell populations in humanized mice peripheral blood over time**

|  | Human immune cells (in % out of hCD45^+^ cells) | | | | |
| --- | --- | --- | --- | --- | --- |
|  | hCD19^+^ | hCD4^+^ | hCD8^+^ | hCD14^+^ | hCD56^+^ |
| Week 7 | 97.4% | 0.4% | 0.5% | 1.6% | 0.2% |
| Week 10 | 96.3% | 0.9% | 0.8% | 4.2% | 0.6% |
| Week 17 | 82.4% | 7% | 5.2% | 4.4% | 0.5% |
| Week 20 | 36.9% | 34.9% | 12% | 8.9% | 0.9% |

**Supplementary Table 2. Significantly differentially expressed genes in pairwise comparisons**

| **Group in which genes are expressed** | **Group of comparison** | **Number of UP genes** | **Number of DOWN genes** | **Top 5 UP genes** | **Top 5 DOWN genes** |
| --- | --- | --- | --- | --- | --- |
| 3x8 Gy + Saline | Saline | 1478 | 2027 | \| KIF1C CHD3  GUCY1A2  TLCD2  GAS8 \| \| --- \| | \| WARS1  RNU2-2P  BMP2K  HLA-DMA  B2M \| \| --- \| |
| 3x8 Gy + Saline dist | Saline | 1439 | 1981 | \| KIF1C  CHD3  GUCY1A2  TLCD2  NBPF1 \| \| --- \| | \| RNU2-2P  WARS1  BMP2K  B2M  HLA-DMA \| \| --- \| |
| 3x8 Gy + Pembro | Saline | 1277 | 2179 | KIF1C  GUCY1A2  NECTIN4 CHD3  PRUNE2 | B2M  WARS1  GBP4  RNU2-2P  STAT1 |
| 3x8 Gy + Pembro dist | Saline | 1322 | 2089 | KIF1C  GUCY1A2 CHD3  PRUNE2  TLCD2 | B2M  WARS1 RNU2-2P  GBP4  STAT1 |
| 3x8 Gy + Saline dist | 3x8 Gy + Saline | 2 | 5 | \| GVINP1  FAT2 \| \| --- \| | \| IGF2  ABI3BP  LINC03040  NID2  LRAT \| \| --- \| |
| 3x8 Gy + Pembro dist | 3x8 Gy + Pembro | 15 | 16 | \| DIO2  LDLRAD4  FAT2  SPARC  NRP2 \| \| --- \| | \| NID2  RYR2 CEACAM1  SGPP2  LINC03040 \| \| --- \| |
| 3x8 Gy + Pembro | 3x8 Gy + Saline | 7 | 114 | \| GVINP1  RYR2  OASL  B3GNT3  LRAT \| \| --- \| | \| XAF1  DIO2  HCP5  HLA-B  GBP4 \| \| --- \| |
| 3x8 Gy + Pembro dist | 3x8 Gy + Saline dist | 0 | 28 | / | \| HCP5  XAF1  NLRC5  HLA-B  GBP4 \| \| --- \| |

**Supplementary Table 3.** **Significantly differentially expressed pathways in pairwise comparisons**

| **Group in which pathways are expressed** | **Group of comparison** | **Pathways UP** | **Pathways DOWN** |
| --- | --- | --- | --- |
| 3x8 Gy + Saline | Saline | -Myogenesis | -Allograft Rejection  -IFN-α Response  -IFN-γ Response  -Complement  -Inflammatory Response  -IL6 JAK STAT3 Signaling  -IL2 STAT5 Signaling  -KRAS Signaling Up  -TNF-α Signaling Via NKκB  -Coagulation  -Apoptosis  -PI3K AKT MTOR Signaling  -ROS Pathway  -MTORC1 Signaling  -Heme Metabolism  -Xenobiotic Metabolism  -P53 Pathway  -Protein Secretion  -G2M Checkpoint  -Fatty Acid Metabolism  -Apical Junction  -KRAS Signaling Dn  -Oxidative Phosphorylation  -E2F Targets |
| 3x8 Gy + Saline dist | Saline | -Myogenesis | -Allograft Rejection  -IFN-γ Response  -IFN-α Response  -Complement  -IL6 JAK STAT3 Signaling  -Inflammatory Response  -IL2 STAT5 Signaling  -KRAS Signaling Up  -TNF-α Signaling Via NKκB  -Coagulation  -Apoptosis  -PI3K AKT MTOR Signaling  -MTORC1 Signaling  -Heme Metabolism  -ROS Pathway  -Apical Junction  -P53 Pathway  -Protein Secretion  -G2M Checkpoint  -Xenobiotic Metabolism  -Cholesterol Homeostasis  -E2F Targets  -Hypoxia  -Fatty Acid Metabolism |
| 3x8 Gy + Pembro | Saline | / | -Allograft Rejection  -IFN-γ Response  -IFN-α Response  -Complement  -Inflammatory Response  -IL6 JAK STAT3 Signaling  -IL2 STAT5 Signaling  -KRAS Signaling Up  -Coagulation  -TNF-α Signaling Via NKκB  -Apoptosis  -ROS Pathway  -PI3K AKT MTOR Signaling  -Heme Metabolism  -Apical Junction  -P53 Pathway  -MTORC1 Signaling  -Fatty Acid Metabolism  -Xenobiotic Metabolism |
| 3x8 Gy + Pembro dist | Saline | / | -Allograft Rejection  -IFN-α Response  -IFN-γ Response  -Complement  -IL6 JAK STAT3 Signaling  -Inflammatory Response  -IL2 STAT5 Signaling  -KRAS Signaling Up  -Coagulation  -TNF-α Signaling Via NKκB  -Apoptosis  -PI3K AKT MTOR Signaling  -ROS Pathway  -MTORC1 Signaling  -P53 Pathway  -Heme Metabolism  -Apical Junction  -KRAS Signaling Dn  -Xenobiotic Metabolism  -Fatty Acid Metabolism |
| 3x8 Gy + Saline dist | 3x8 Gy + Saline | / | / |
| 3x8 Gy + Pembro dist | 3x8 Gy + Pembro | / | / |
| 3x8 Gy + Pembro | 3x8 Gy + Saline | -MYC Targets  -Oxidative Phosphorylation  -Hypoxia  -Glycolysis  -MTORC1 Signaling  -Adipogenesis  -Protein Secretion  -Unfolded Protein Response  -Cholesterol Homeostasis  -Fatty Acid Metabolism  -Estrogen Response Early  -Peroxisome  -Estrogen Response Late  -P53 Pathway  -Heme Metabolism | -IFN-γ Response  -Allograft Rejection  -IL6 JAK STAT3 Signaling  -IFN-α Response  -Inflammatory Response  -Complement  -KRAS Signaling Up  -Coagulation  -IL2 STAT5 Signaling  -Bile Acid Metabolism  -Hedgehog Signaling  -TNF-α Signaling Via NKκB  -Apical Junction |
| 3x8 Gy + Pembro dist | 3x8 Gy + Saline dist | -MYC Targets  -G2M Checkpoint  -Hedgehog Signaling  -Protein Secretion  -NOTCH Signaling | -Allograft Rejection  -IFN-γ Response  -Apical Junction  -Peroxisome  -KRAS Signaling Dn  -IFN-α Response |
