# Supplementary material for: Immuno-radiotherapy enhances tumor control and induces abscopal responses in a humanized mouse model
Source: Front Immunol. 2026 Mar 11;17:1774955. doi: 10.3389/fimmu.2026.1774955 (PMC13013455; doi:10.3389/fimmu.2026.1774955)

Suppl Figure 1

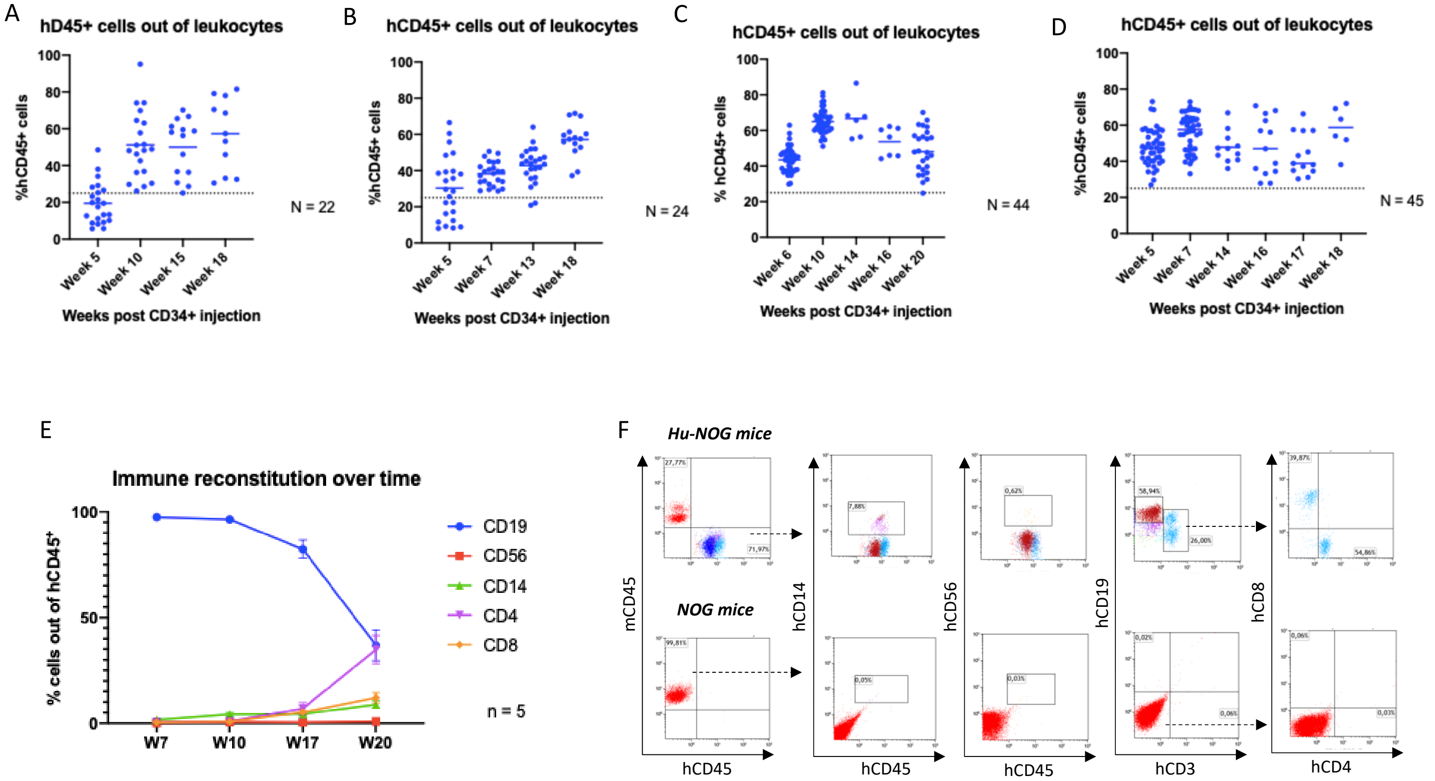

Suppl Figure 2

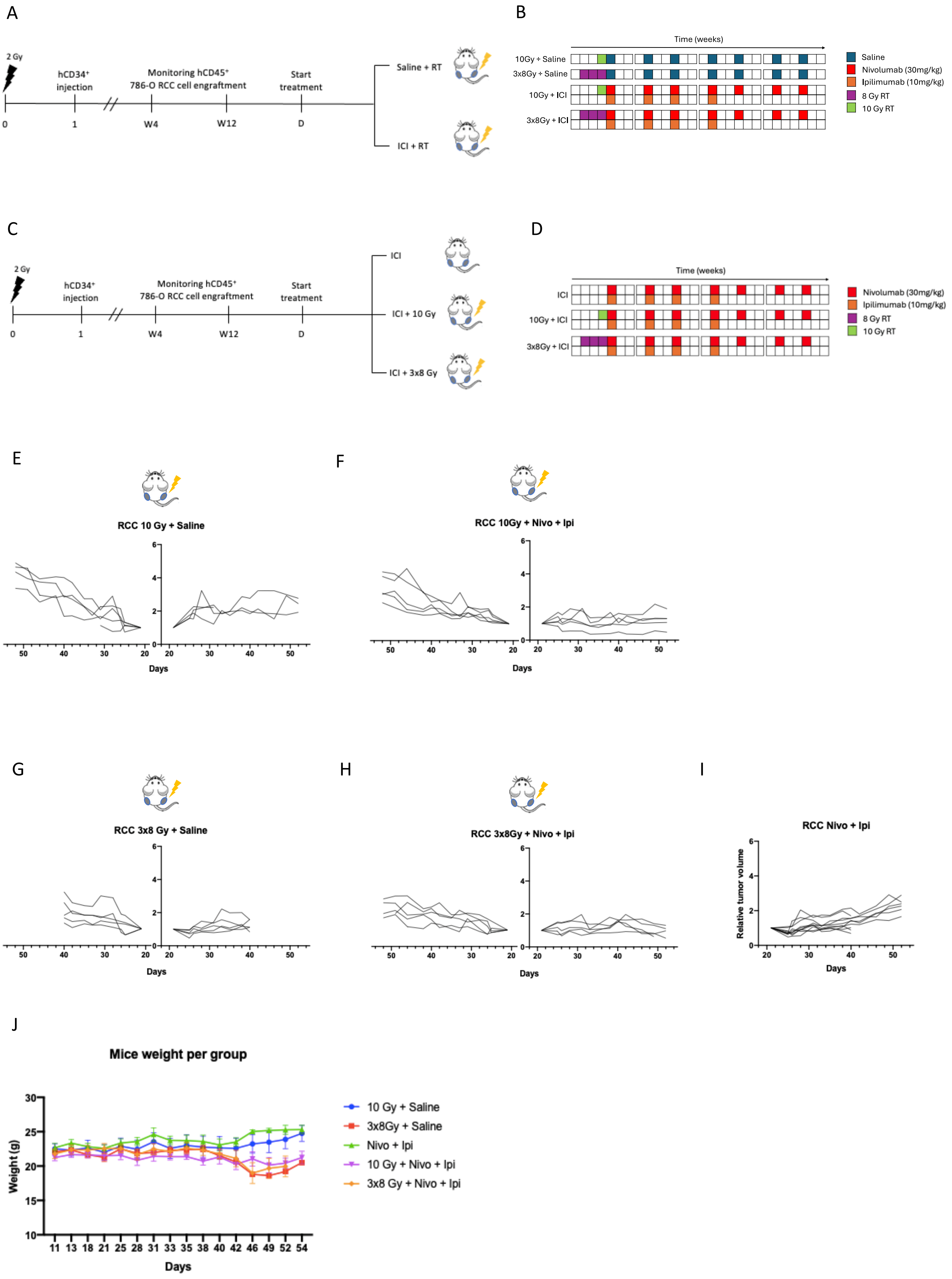

Suppl Figure 3

A

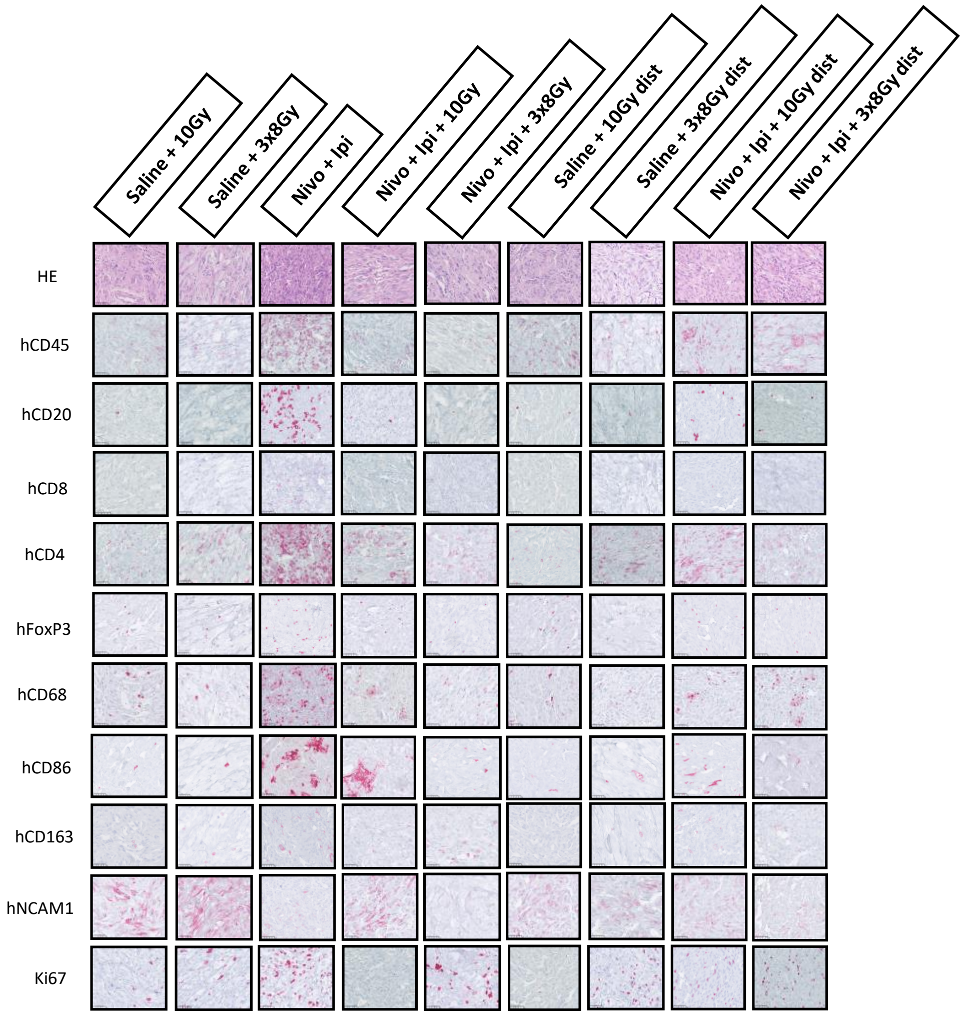

B

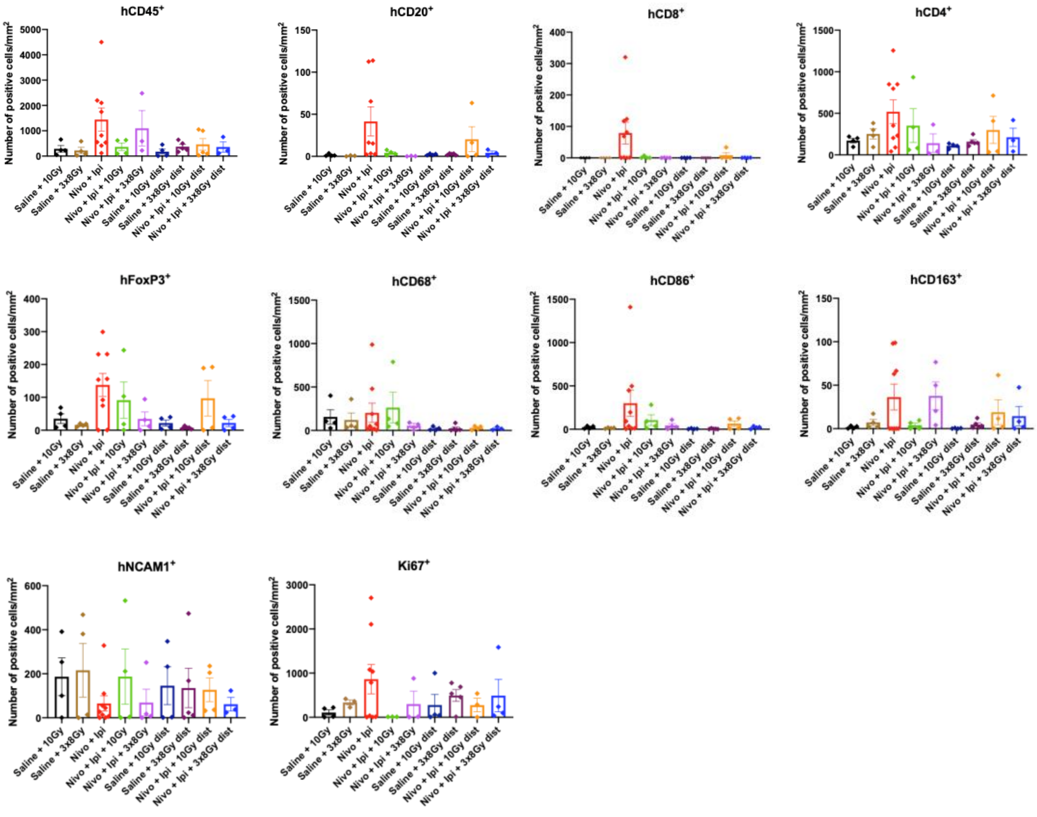

Suppl Figure 4

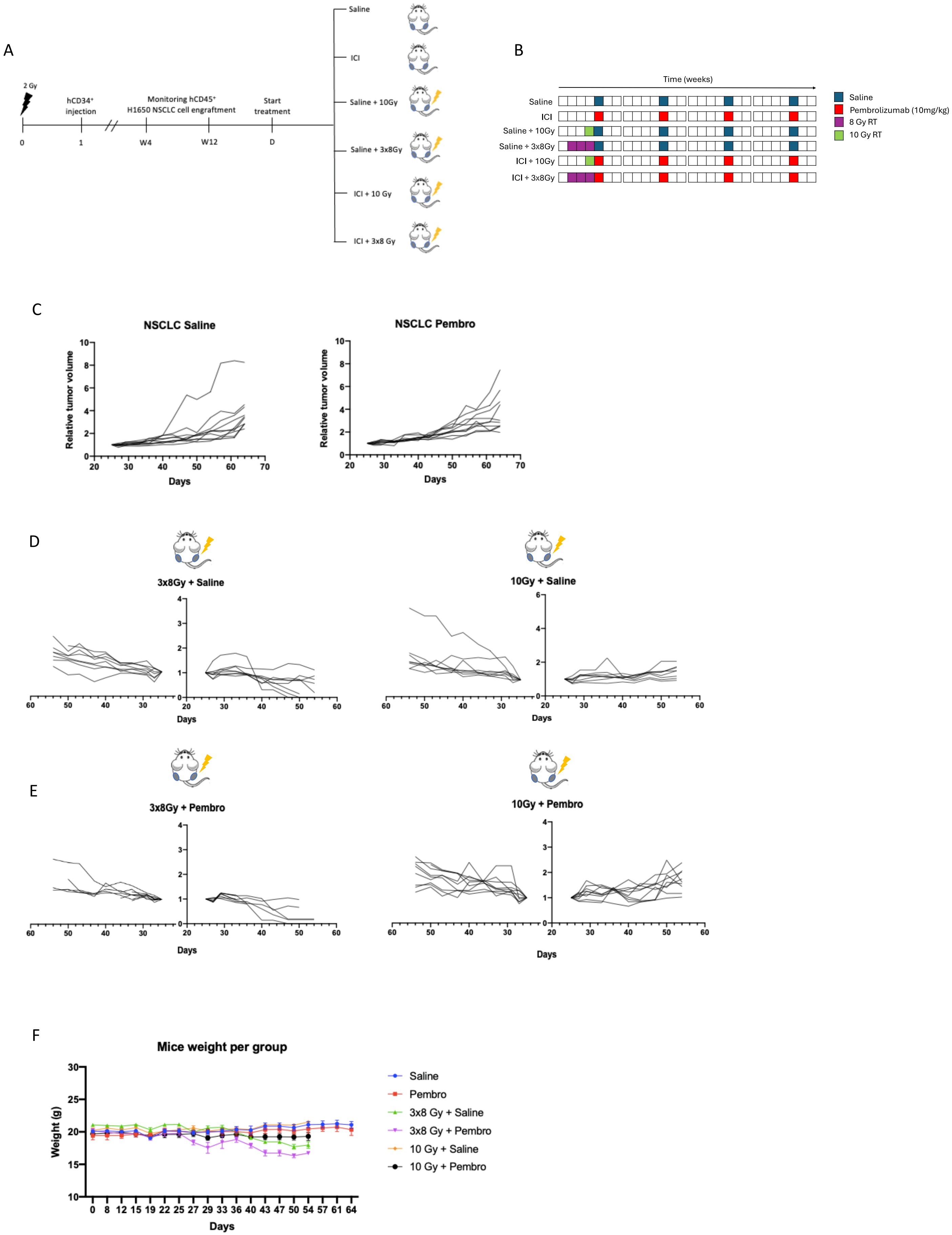

Suppl Figure 5

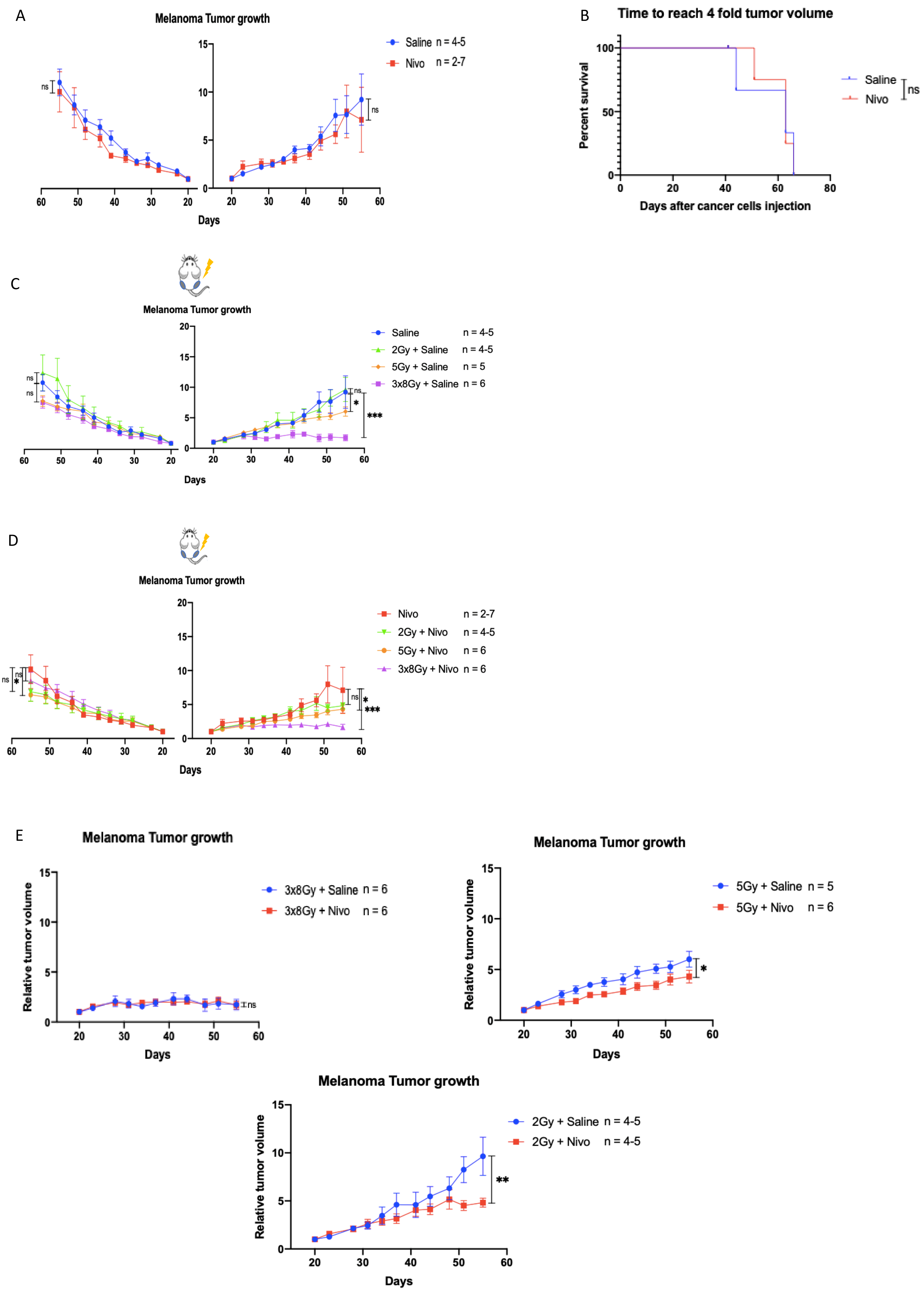

Suppl Figure 6

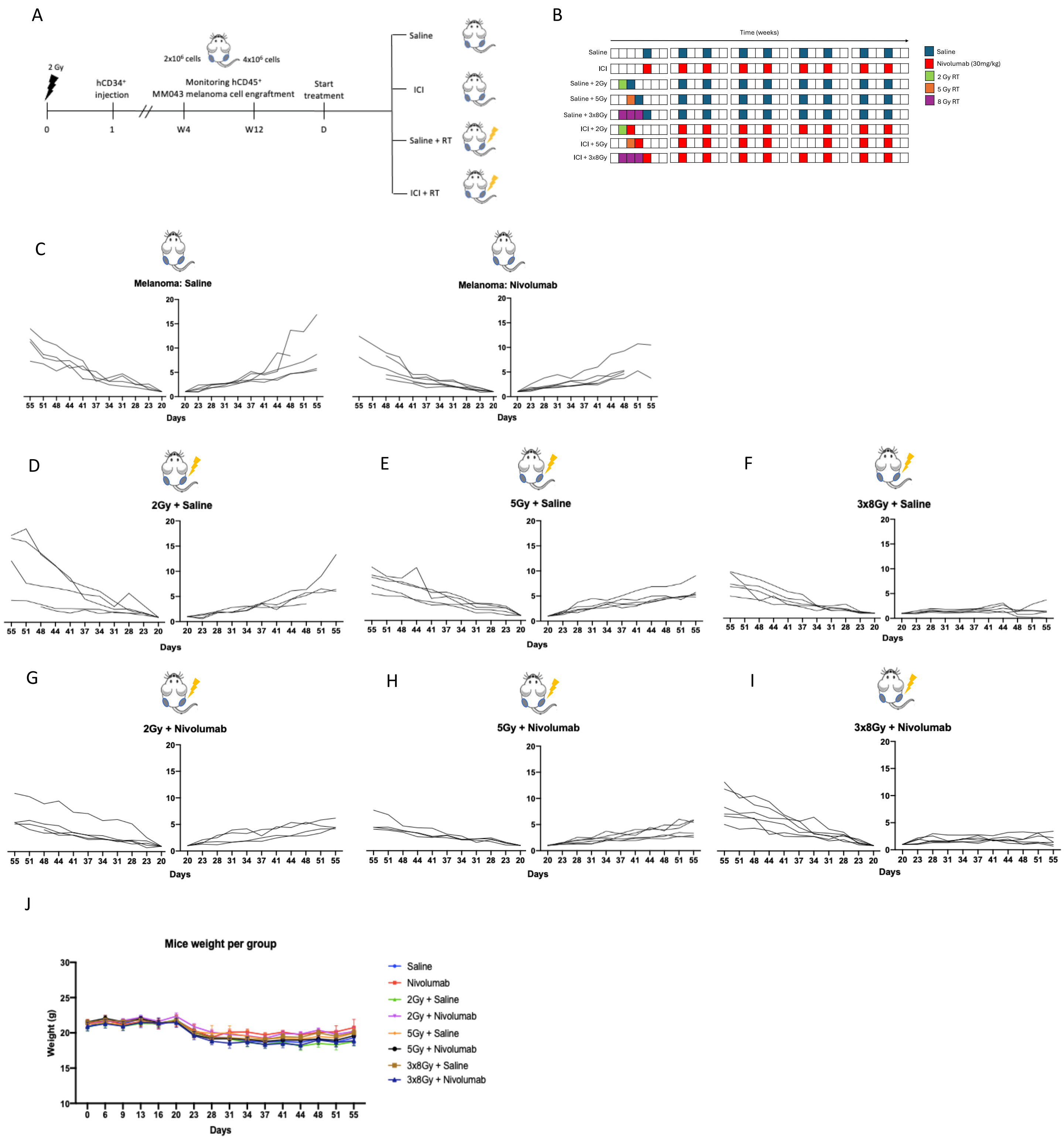

Suppl Figure 7

A

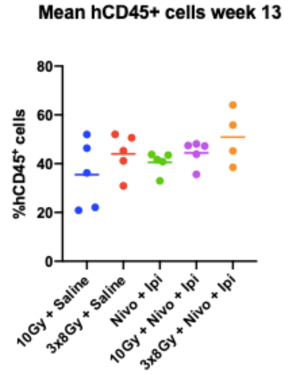

B

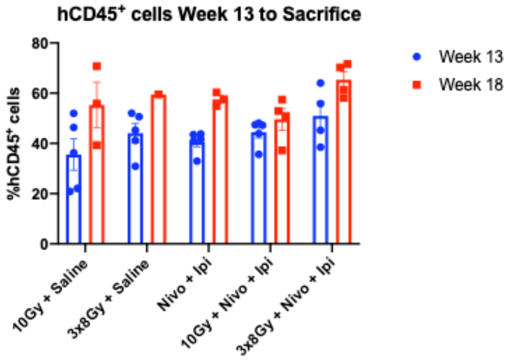

C

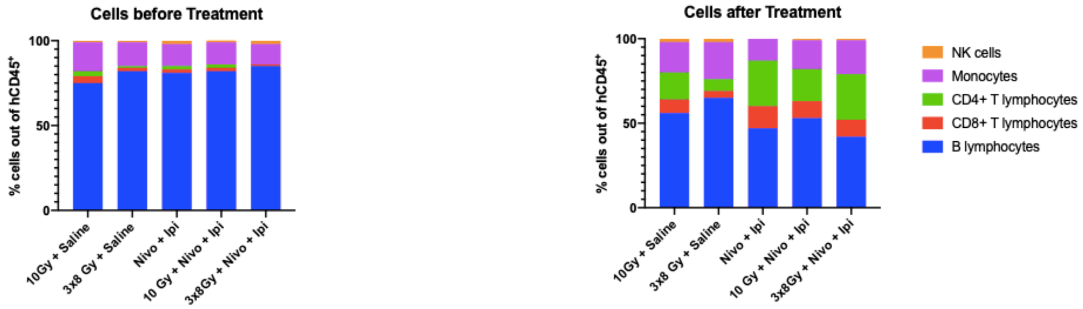

D

	B Lymphocytes				Monocytes				NK cells				CD4+ T cells				CD8+ T cells			
	3x8 Gy + Saline	Nivo + Ipi	10 Gy + Nivo + Ipi	3x8 Gy + Nivo + Ipi	3x8 Gy + Saline	Nivo + Ipi	10 Gy + Nivo + Ipi	3x8 Gy + Nivo + Ipi	3x8 Gy + Saline	Nivo + Ipi	10 Gy + Nivo + Ipi	3x8 Gy + Nivo + Ipi	3x8 Gy + Saline	Nivo + Ipi	10 Gy + Nivo + Ipi	3x8 Gy + Nivo + Ipi	3x8 Gy + Saline	Nivo + Ipi	10 Gy + Nivo + Ipi	3x8 Gy + Nivo + Ipi
10 Gy + Saline	0.89	0.41	0.97	0.02	0.99	0.74	1.00	>1.00	1.00	0.17	0.7	0.00	0.73	0.3	0.96	0.42	0.78	0.29	0.98	0.99
3x8 Gy + Saline		0.22	0.64	0.02		0.71	0.97	0.99		0.68	0.98	0.14		0.09	0.43	0.13		0.11	0.52	0.59
Nivo + Ipi			0.73	0.56			0.85	0.74			0.76	0.54			0.6	1.00			0.53	0.43
10 Gy + Nivo + Ipi				0.04				1.00				0.05				0.76				1.00

E

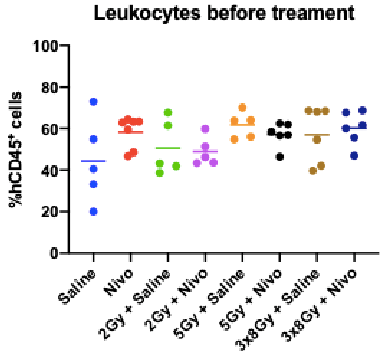

F

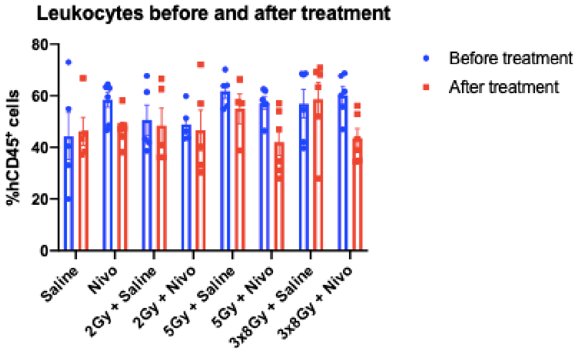

G

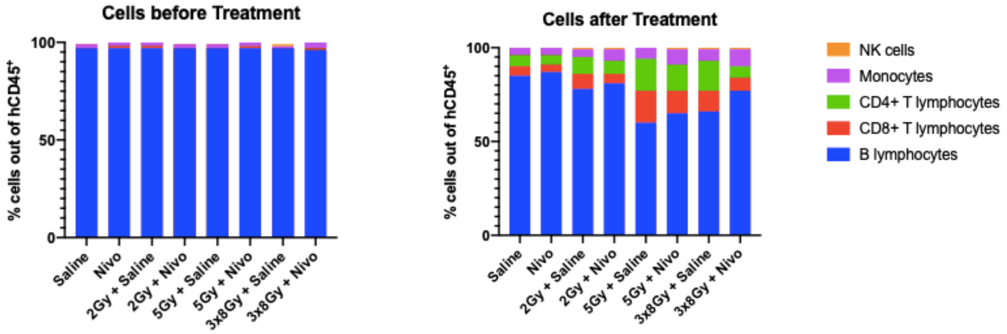

H

	B Lymphocytes							Monocytes						
	Nivo	2 Gy + Saline	2 Gy + Nivo	5 Gy + Saline	5 Gy + Nivo	3x8 Gy + Saline	3x8 Gy + Nivo	Nivo	2 Gy + Saline	2 Gy + Nivo	5 Gy + Saline	5 Gy + Nivo	3x8 Gy + Saline	3x8 Gy + Nivo
Saline	0.85	1.00	>1.00	0.80	0.16	0.08	>1.00	>1.00	>1.00	0.69	0.53	0.10	0.73	0.00
Nivo		0.47	0.92	0.08	0.00	0.00	0.71		>1.00	0.47	0.33	0.03	0.50	0.00
2 Gy + Saline			1.00	0.98	0.46	0.28	1.00			0.72	0.56	0.11	0.76	0.00
2 Gy + Nivo				0.71	0.11	0.05	>1.00				>1.00	0.96	>1.00	0.20
5 Gy + Saline					0.98	0.93	0.85					1.00	1.00	0.47
5 Gy + Nivo						>1.00	0.17						0.90	0.80
3x8 Gy + Saline							0.08							0.11

	NK cells							CD4+ T cells							CD8+ T cells						
	Nivo	2 Gy + Saline	2 Gy + Nivo	5 Gy + Saline	5 Gy + Nivo	3x8 Gy + Saline	3x8 Gy + Nivo	Nivo	2 Gy + Saline	2 Gy + Nivo	5 Gy + Saline	5 Gy + Nivo	3x8 Gy + Saline	3x8 Gy + Nivo	Nivo	2 Gy + Saline	2 Gy + Nivo	5 Gy + Saline	5 Gy + Nivo	3x8 Gy + Saline	3x8 Gy + Nivo
Saline	0.99	0.48	0.99	0.17	0.75	0.40	0.00	>1.00	1.00	1.00	0.75	0.27	0.19	>1.00	1.00	0.92	1.00	0.32	0.01	0.17	0.99
Nivo		0.08	0.69	0.02	0.20	0.05	<0.00		0.98	0.98	0.51	0.09	0.06	>1.00		0.62	0.94	0.11	0.00	0.03	0.82
2 Gy + Saline			0.94	1.00	1.00	>1.00	0.31			>1.00	0.95	0.61	0.48	1.00			1.00	0.92	0.26	0.88	>1.00
2 Gy + Nivo				0.62	1.00	0.92	0.02				0.96	0.69	0.55	1.00				0.73	0.11	0.62	>1.00
5 Gy + Saline					0.92	1.00	0.86					>1.00	1.00	0.67					0.99	>1.00	0.75
5 Gy + Nivo						1.00	0.08						>1.00	0.17						0.95	0.08
3x8 Gy + Saline							0.28							0.12							0.61

Suppl Figure 8

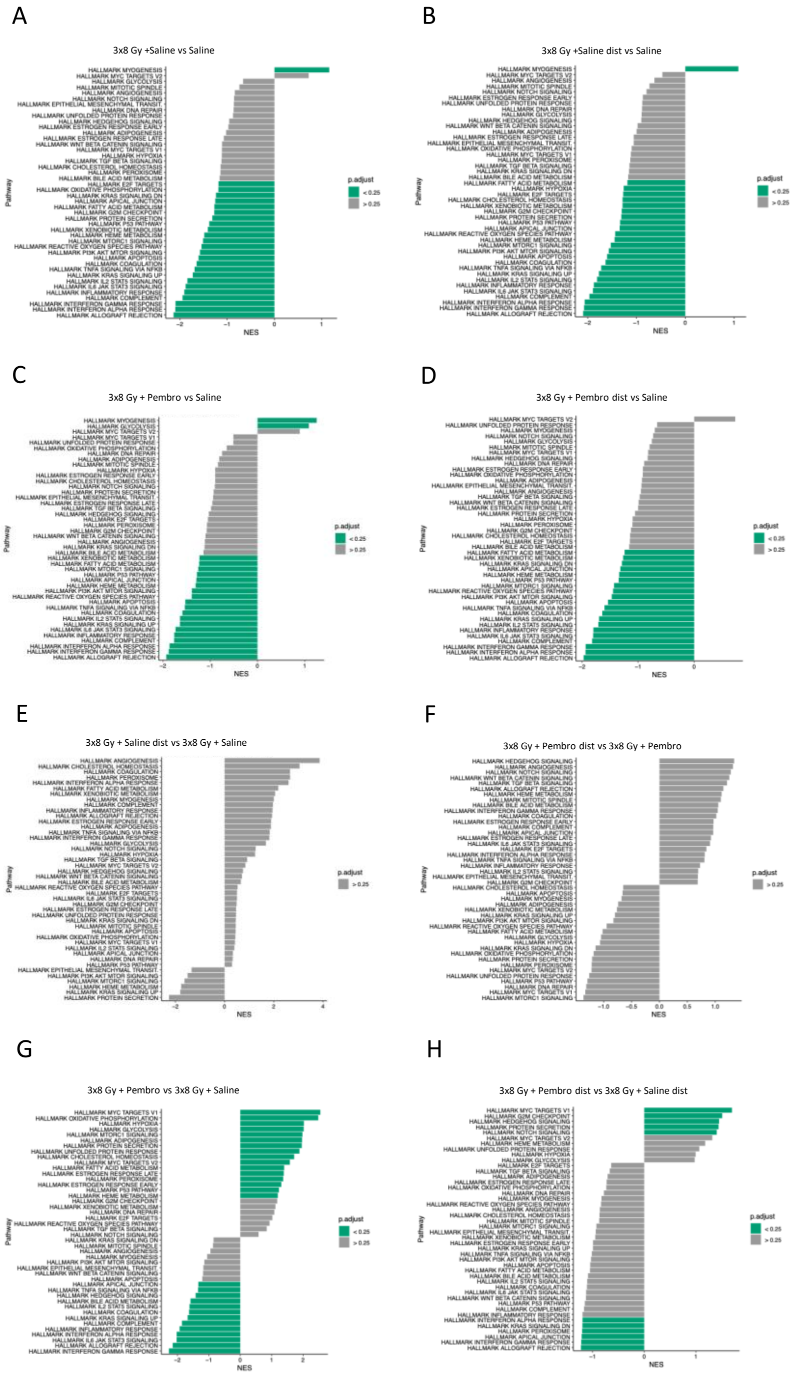

Suppl Figure 9

A

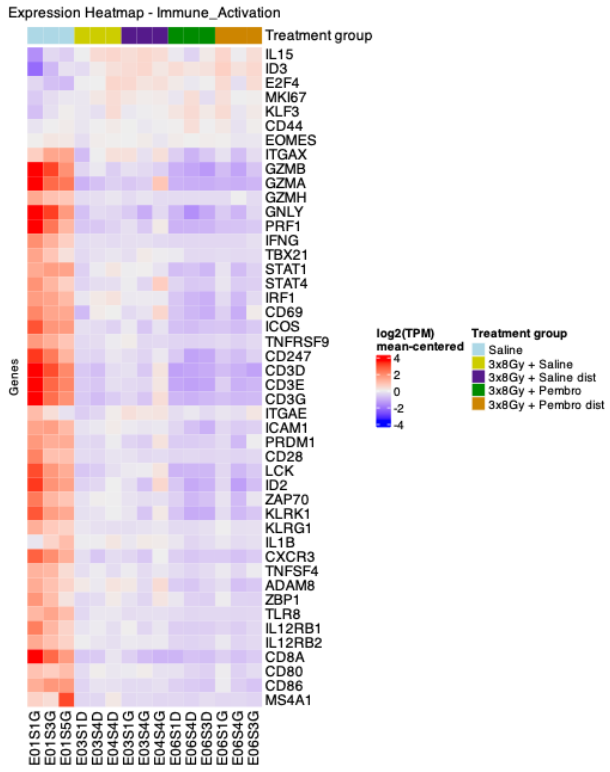

Supplement: Supplementary Figure 1 — (A–D). Flow cytometry quantification of human CD45+ cells out of all leukocytes in humanized mice peripheral blood over time in individual experiments. (n = 22-44). Mice were myeloablated by sublethal irradiation before HSC engraftment (E). Flow cytometry quantification of human CD19+, CD56+, CD14+, CD4+ and CD8+ cells out of human CD45+ cells over time. (n = 5). (F). Representative flow cytometry plots identifying human immune subpopulations in peripheral blood of humanized and non-humanized mice. All data represent either individual values with mean or mean +/- SEM. [file DataSheet1.pdf]
